# Expression profiles of the autism-related SHANK proteins in the human brain

**DOI:** 10.1186/s12915-023-01712-0

**Published:** 2023-11-13

**Authors:** Sarah Woelfle, Maria T. Pedro, Jan Wagner, Michael Schön, Tobias M. Boeckers

**Affiliations:** 1 Institute for Anatomy and Cell Biology, Ulm University, Albert-Einstein-Allee 11, 89081 Ulm, Germany; 2 Department of Neurosurgery, Ulm University, Campus Günzburg, Lindenallee 2, 89312 Günzburg, Germany; 3 Department of Neurology, Ulm University and Universitäts- and Rehabilitationskliniken Ulm, 89081 Ulm, Germany; 4 Deutsches Zentrum Für Neurodegenerative Erkrankungen, DZNE, Ulm Site, 89081 Ulm, Germany

**Keywords:** SHANK2, SHANK3, *Post mortem* human brain, Brain regions, Protein expression, Autism spectrum disorders

## Abstract

**Background:**

SHANKs are major scaffolding proteins at postsynaptic densities (PSDs) in the central nervous system. Mutations in all three family members have been associated with neurodevelopmental disorders such as autism spectrum disorders (ASDs). Despite the pathophysiological importance of SHANK2 and SHANK3 mutations in humans, research on the expression of these proteins is mostly based on rodent model organisms.

**Results:**

In the present study, cellular and neuropil SHANK2 expression was analyzed by immunofluorescence (IF) staining of *post mortem* human brain tissue from four male individuals (19 brain regions). Mouse brains were analyzed in comparison to evaluate the degree of phylogenetic conservation. Furthermore, SHANK2 and SHANK3 isoform patterns were compared in human and mouse brain lysates. While isoform expression and subcellular distribution were largely conserved, differences in neuropil levels of SHANK2 were found by IF staining: Maximum expression was concordantly measured in the cerebellum; however, higher SHANK2 expression was detected in the human brainstem and thalamus when compared to mice. One of the lowest SHANK2 levels was found in the human amygdala, a moderately expressing region in mouse. Quantification of SHANK3 IF in mouse brains unveiled a distribution comparable to humans.

**Conclusions:**

In summary, these data show that the overall expression pattern of SHANK is largely conserved in defined brain regions; however, differences do exist, which need to be considered in the translation of rodent studies. The summarized expression patterns of SHANK2 and SHANK3 should serve as a reference for future studies.

**Supplementary Information:**

The online version contains supplementary material available at 10.1186/s12915-023-01712-0.

## Background

ProSAP/SHANKs are multidomain scaffold proteins found at postsynaptic densities (PSDs) of excitatory synapses with a crucial impact on synaptic plasticity [1–4]. The indispensable role of SHANKs in human brains was first revealed by the causative role of *SHANK3* haploinsufficiency in Phelan-McDermid syndrome (PMDS) [5, 6], “a syndromic form of autism spectrum disorders (ASD)” [7].

*Shank2* is enriched in the brain and moderately expressed in extra-neuronal tissues such as the human and rodent liver and kidney [8, 9]. Within the rat brain, SHANK2 protein is widely expressed, for example in the cerebral cortex, cerebellum (molecular layer), hippocampus, thalamus, and basal ganglia [10]. Subcellularly, SHANK2 is localized at PSDs of excitatory synapses [10], but recent studies also indicate an axonal role [11, 12] in rodents. Alternative splicing and intragenic promoters produce a variety of isoforms, which led to the initial description of three major SHANK2 isoforms that are present also in humans: SHANK2E represents the largest isoform, which owes its name to the source where it was first identified (“E” for epithelial cells) [13]. SHANK2A lacks the ankyrin repeats domain (ANK), while isoform SHANK2 is composed of the Src homology 3 (SH3), PSD-95/Discs large/zonula occludens-1 (PDZ), proline-rich clusters (PRC), and sterile alpha motif (SAM) domains [9]. The existence of a more complex isoform machinery is not unlikely and first evidence has been provided (human isoform AF141901 [9] and [14]). Alterations in *SHANK2* have been identified in patients with ASD (e.g., [15]), intellectual disability (ID, e.g., [16]), and schizophrenia (e.g., [17]). Consequently, a few years later, *Shank2*^*−/−*^ mouse models revealed “autistic-like behavior” as well as biochemical and electrophysiological alterations at synapses [18, 19]. Cell type-specific deletions of *Shank2* in the Purkinje cells of the cerebellum [20, 21], in all parvalbumin-positive cells [22], and in CAMKIIA- and Viaat-positive excitatory and inhibitory neurons [23] reflected distinct features of an autism-related phenotype.

Despite the relevance of SHANK2 in human neurological disorders and the link between cellular SHANK2 expression and behavioral phenotypes, protein expression studies have been largely performed in rodents. First, the anatomical differences between the human and mouse brain must be taken into account, as central regions like the transentorhinal cortex or some hypothalamic and brainstem nuclei [24] are not present in mice. In addition, cellular characteristics are disparate [25, 26]. Second, recent transcriptomic [27, 28] and proteomic [29] studies reported pronounced differences between humans and mice including Alzheimer’s disease (AD)- and autism-related genes [28], thus underscoring the necessity of studies validating protein expression patterns in humans. An important contribution to this undertaking has been made by the online resource “The Human Protein Atlas” (HPA) [30]. However, for the SHANK proteins, protein expression is annotated maximum for four regions and expression was determined semi-quantitatively in single chromogenic stainings.

Therefore, the aim of this study was to analyze neuropil and cellular SHANK2 expression in core regions of* post mortem *human brains. Intensity and density of SHANK2-positive puncta in the neuropil was measured in a total of 19 different brain regions (*n* = 4 individuals) by high-resolution confocal microscopy and compared to mouse brains which were analyzed identically. In contrast to destructive biochemical approaches preceding proteomics or western blots, three-dimensional (3D) tissue immunofluorescence (IF) staining has the advantage of analyzing the precise location of a protein and, hence, was used to study (sub-)cellular SHANK2 expression. In addition, using commercially available human brain lysates, the aim was to assess conservation of SHANK2 isoforms. To complement an existing semi-quantitative study on SHANK3 in human brains [31], neuropil SHANK3 expression was measured in mouse brain sections and brain lysates. The results were summarized in brain-wide heat maps of protein expression.

## Results

### Characterization of a specific SHANK2 antibody for use on human tissue and its pattern in IF

To study SHANK2 protein expression in the human brain, we first aimed to identify an antibody that specifically detects our target protein in *post mortem* tissue using a defined battery of experiments. We eventually chose a commercial, polyclonal SHANK2 antibody (raised against a part of the human protein, as shown in Additional file 1a; previously used in “The HPA”) that met our criteria as described below.

**Fig. 1:**
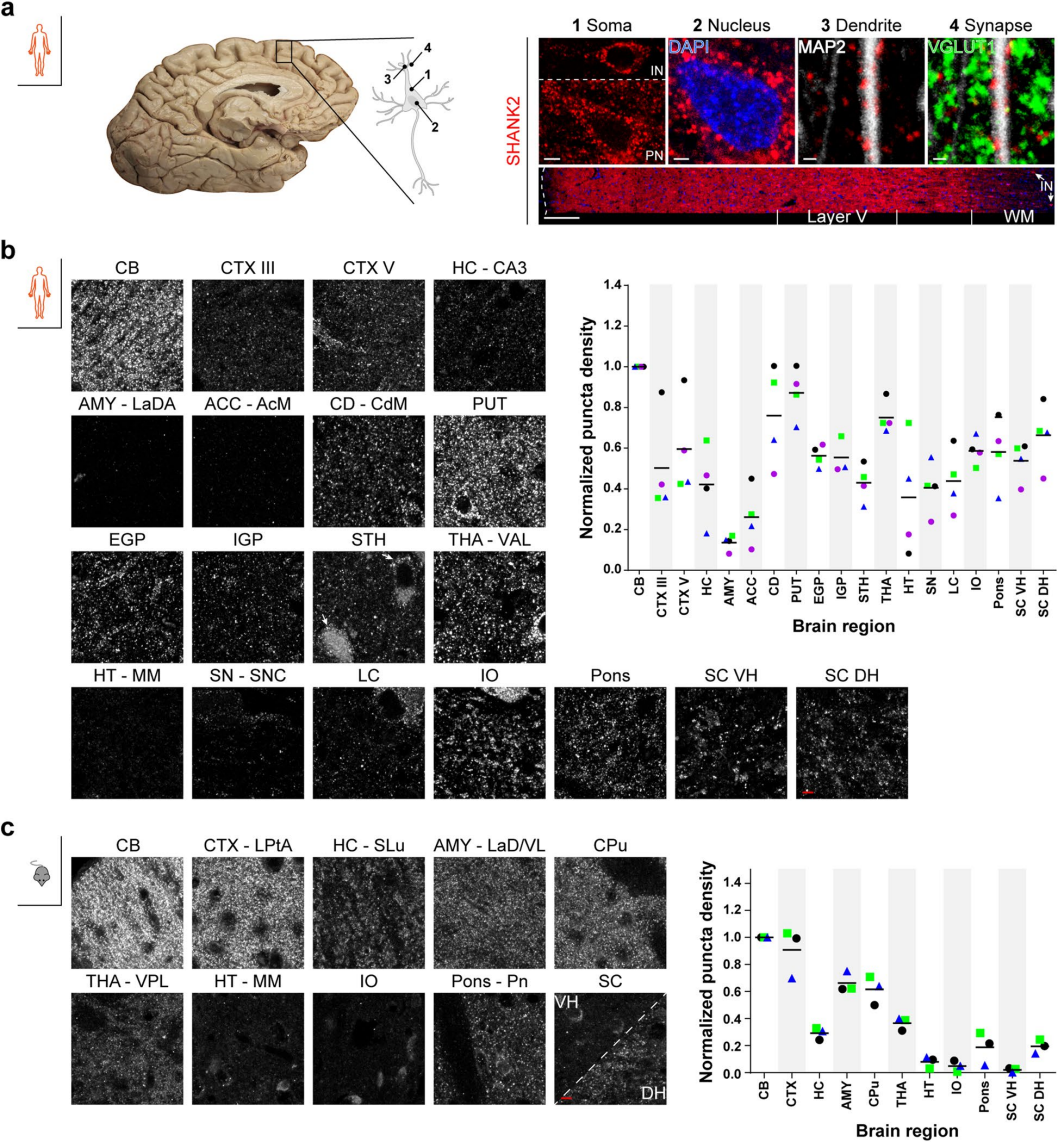
SHANK2 expression in core human and mouse brain regions. **a** Staining pattern of the selected SHANK2 antibody (Sigma) in a superior frontal gyrus (SFG) section from a *post mortem* human brain. Scale bars, *1*, 5 µm, *2*, 2 µm, *3* and *4*, 1 µm. The tilescan (bottom, right) shows the six cortical layers and the underlying white matter (WM). The outer border of the tissue section is traced by a dashed white line. SHANK2-positive interstitial neurons (INs) are marked by arrows; the lower neuron is shown in higher magnification in *1*. Scale bar, 200 µm. Neuronal sketch (left) adapted from [33]. PN = pyramidal neuron. **b** Representative images of human SHANK2 protein expression (case 2, purple in the graph) in all analyzed subregions. Arrows in the STH image highlight SHANK2 IF in neurons. Scale bar, 5 µm (left). The normalized mean SHANK2 puncta density in the neuropil is plotted on the right (*n* = 4). The IGP of case 4 was not discernible by eye. **c** Representative images of mouse SHANK2 protein expression (blue triangle in the graph) in all analyzed subregions. Scale bar, 5 µm (left). The normalized mean SHANK2 puncta density in the neuropil is plotted on the right (*n* = 3). In **b** and **c**, values were normalized to the CB; means of the analyzed *n* are indicated by horizontal lines. Brightness/contrast adjustments were performed identically for all regions in human and mouse, respectively

In a frontal cortex section from a male individual (case 2, [32] and Table 1), SHANK2-positive puncta were found in the soma of pyramidal neurons and in the neuropil, while the nuclei were devoid of SHANK2 IF (Fig. 1a, 1 PN and 2). The staining pattern was validated in a tissue resectate from a young epilepsy patient (21 years at surgery); importantly, this tissue is characterized by minimal fixation delay (Additional file 1b). Co-staining experiments demonstrated SHANK2 signal along dendrites and in close apposition to presynaptic vesicular glutamate transporter 1 (VGLUT1) (Fig. 1a, 3, and 4), providing proof-of-concept. A tilescan covering all six neocortical layers and the underlying white matter (WM, Fig. 1a, lower row) showed concerted expression in the gray matter (highest intensity values in layer V), with nearly absent signal in the white matter, apart from somatic SHANK2 in interstitial neurons (Fig. 1a, 1 IN and arrows in tilescan). These results were consistent with the expected expression pattern of a mainly postsynaptic protein. Secondly, the cross-reactivity against SHANK1 and SHANK3 was assessed using recombinant human proteins in western blots. Only after incubation with recombinant human SHANK2 protein did the selected SHANK2 antibody show a signal between 140 and 260 kDa (Additional file 1c), which was also present when staining against the mCherry tag. To confirm successful protein overexpression, the membrane containing recombinant SHANK1 and SHANK3 was incubated with antibodies directed against these proteins. Now, signal was obtained at the expected heights (Additional file 1c, outer right) in two technical replicates. In summary, it was shown that the selected antibody specifically detects SHANK2.

**Table 1:** Demographics of study cases

**Case**	**Sex**	**Age**	**Cause of death**	**AD stage (NFT/Aβ)**	**PD stage**	**PMI (hours)**	**Fixation year**
1	Male	71	Cardiogenic shock	I/0	0	72	2019
2	Male	74	Decompensated heart insufficiency	I/3	0	24	2017
3	Male	89	Myocardial infarct	I/3	1	24	2016
4	Male	77	Global respiratory insufficiency	I/0	0	48	2016
5	Female	21^a^	-	-	-	Fixation delay ~ 45 min	2019

*AD* Alzheimer’s disease, *Aβ* amyloid-β, *PD* Parkinson’s disease, *PMI* *post mortem* interval

^a^At the time point of surgery

For SHANK2 IF staining, brains from *n* = 4 male individuals at low stages of AD and PD were selected (for case 1–3: see also [32]). Initially, SHANK2 puncta density was measured in the superior frontal gyrus (SFG) to get an idea on the comparability of the cases, which was given for the analyzed layers III and V (Additional file 1d). Additionally, a mean of 1.36 SHANK2 puncta per µm of MAP2-positive dendrite was determined, which corresponds to the aged human cingulate cortex (mean spine density at apical and basal dendrites = 1.367, 85-year-old man, see [34]) (Additional file 1e).

Contrary to the prediction from “The HPA” (“Western blot: only bands not corresponding to the predicted size.” [35]), the SHANK2 antibody produced a specific signal in our hands (Additional file 1c). It was tested on human, mouse, and rat cortical lysates and compared to the pattern of a well-established homemade antibody (SA5192, see [10]). Both antibodies detected isoforms SHANK2A and SHANK2 concordantly in the human lysate (Additional file 1f). The commercial SHANK2 antibody unveiled the same pattern in mouse and rat cortical lysates (Additional file 1g), indicating overall reactivity with these species due to the high conservation of the antigenic sequence (see alignment in Additional file 1h). However, in direct comparison, the homemade serum was more sensitive already at a higher dilution (Additional file 1f), which is why this antibody was used for western blot experiments.

### SHANK2 expression in the neuropil of core human brain regions

Before performing SHANK2 IF staining, sections from all tissue blocks were incubated either with the secondary antibody alone (Additional file 2c, upper row) or stained after incubation with anti-rabbit IgG to serve as a “pure” isotype control (Additional file 2c, lower row). For both conditions, no signal other than autofluorescent perinuclear lipofuscin was observed. In the granular layer of the cerebellum (CB), lipofuscin was largely scattered in granules (see also [36]), in contrast to the vine-like arrangement around nuclei in most other regions (Additional file 2c, inset). Bright, intracellular SHANK2 puncta could be distinguished from lipofuscin (pale, larger in diameter), which was relevant for later analysis of cellular SHANK2 expression (Additional file 2d). Staining and secondary only control with identical acquisition and contrast settings are opposed in Additional file 2e and were part of each IF experiment.

A detailed analysis of SHANK2 puncta in the neuropil was performed in 19 human brain regions (see examples in Additional file 2a-b) and eleven mouse brain regions. The protein expression data presented below reflect the respective subregions’ SHANK2 expression (see Table S1 for an overview), not the entire brain region. For simplicity, subregions are only described at first mention; thereafter, the abbreviation of the superordinate brain region is used (column 2, Table S1).

The quantitative analysis of neuropil/synaptic SHANK is based on the detection of SHANK-immunoreactive 3D surfaces (for details, see “Methods”) that are referred to as “puncta.” The results of SHANK puncta analysis are reported by three measures: Puncta density compares the density of SHANK-containing synapses (puncta number/ROI (3D tissue volume in neuropil)). Sum intensity is used as a parameter for the amount of protein at the presumable synaptic punctum (protein amount (fluorescence signal) in a punctum). To use it as parameter for the SHANK expression level in a given region, it is reported as the sum of protein amounts of all puncta in a defined 3D tissue volume (see “Methods”). Mean intensity reports the mean protein amount of a presumable synaptic punctum and, as such, considers sum intensity and volume of the punctum (protein amount/volume(punctum)). SHANK2 expression was highest in the molecular layer of the CB in all four cases (Table 1). The data from other regions were normalized (0–1) to the CB (representative images: Fig. 1b, left; graph: Fig. 1b, right). In the telencephalon, SHANK2 puncta density in layers III and V of the SFG (CTX III and CTX V) was around 0.5, comparable to the *stratum pyramidale* (CA3) of the hippocampus (HC). The lateral amygdaloid nucleus (AMY) had the lowest puncta density, together with the ventral striatum (medial accumbens nucleus, ACC). In the dorsal striatum, overview tilescans indicated a specific pattern of SHANK2: larger areas of moderate SHANK2 intensity were interspersed with SHANK2-enriched structures (see also “Characteristic SHANK2 expression in sublayers of analyzed brain regions”). Analysis was performed in the latter to get an idea of the upper limit of SHANK2 in these regions. Here, mean SHANK2 puncta density in the medial caudate nucleus (CD) and putamen (PUT) was more than two thirds. Additional regions associated with the basal ganglia system (external and internal segment of the globus pallidus (EGP and IGP), and the subthalamic nucleus (STH)) had moderate SHANK2 puncta density around cortical levels. In the pallidum, SHANK2 puncta strikingly indicated the outline of dendrites (Fig. 1b, left, EGP, IGP and MAP2 co-staining in Additional file 3a-c). Large glutamatergic neurons in the STH were SHANK2-positive (Fig. 1b, left, arrows). In the diencephalon, SHANK2 puncta density in the lateral ventroanterior nucleus of the thalamus (THA) was among the highest values, while the medial mammillary nucleus of the hypothalamus (HT) displayed half the density. In the midbrain, SHANK2 in the neuropil was largely detected along neurites emerging from large, dopaminergic, SHANK2-positive neurons in the substantia nigra, pars compacta (SN). A similar pattern was observed in the locus coeruleus (LC) (see also Additional file 3e). In the medulla oblongata, the principal subnucleus of the inferior olive (IO) was assessed. Intriguingly, fine SHANK2 puncta in the neuropil were assembled in groups reminiscent of glomeruli, which are typical for this brain region (see Fig. 1b, IO). Cellular SHANK2 was also detected. SHANK2 puncta density in the IO and the pontine nuclei (pons) was in the midfield, but one of the highest after striatum and THA. SHANK2 was comparably abundant in the lumbar spinal cord (SC). Common to all cases was a SHANK2 signal in motor neurons (MNs) and along neurites in lamina IX (ventral horn, SC VH). In lamina I (dorsal horn, SC DH), scattered SHANK2 puncta were present in the neuropil.

To determine the relative levels of neuropil/synaptic SHANK2 expression, the sum intensities of all detected puncta in the neuropil were plotted. Following the CB, the sum intensities were highest in the CD, PUT, and THA (Additional file 4a), in accordance with puncta density (Fig. 1b). Weak SHANK2 IF in the striatum had, on average, 15% lower sum intensities in comparison (overview scans, not included in the graph). CTX V, IO, and pons had moderate expression levels (mean = 0.33–0.67). The lowest SHANK2 sum intensities were detected in the AMY and ACC. All other regions had comparable sum intensities, with means between 0.17 and 0.39 (Additional file 4a). In summary, low expression (mean = 0–0.33) was observed in the AMY (mean = 0.03), ACC (mean = 0.11), HC (mean = 0.17), HT (mean = 0.19), SN (mean = 0.25), IGP (mean = 0.29), and CTX (layer III, mean = 0.31). Slightly higher expression was found in the STH (mean = 0.35), the LC (mean = 0.37), and the EGP (mean = 0.39). Moderate expression (mean = 0.33–0.67) was detected in CTX V (mean = 0.40), IO (mean = 0.51), and pons (mean = 0.59). High expression (mean ≥ 0.67) was measured in specific regions of the CD (mean = 0.74) and PUT (mean = 0.92) and in the THA (mean = 0.77).

The mean intensities of SHANK2 puncta in the neuropil demonstrated the least variability on average across most regions (Additional file 4b; exceptions: AMY, CD, PUT, THA, and SC). It is noteworthy that while the mean intensity and puncta density were nearly identical in the IO and the pons, the overall sum intensity was lower in the IO, most likely reflecting the smaller volume of the individual puncta.

### SHANK2 expression in the neuropil of core mouse brain regions

Consistent with human data, SHANK2 expression was highest in the CB (Fig. 1c, left), and expression in other brain regions was normalized to the CB on a scale of 0 to 1. SHANK2 puncta density in the CTX was comparable to the maximum density. Puncta densities in the AMY and CPu were around two thirds of the maximum, and puncta in the HC (specifically, the *stratum lucidum* (SL)), THA, pons, and SC DH were half as dense. HT, IO, and SC VH were almost devoid of SHANK2 puncta (Fig. 1c, right).

**Fig. 2:**
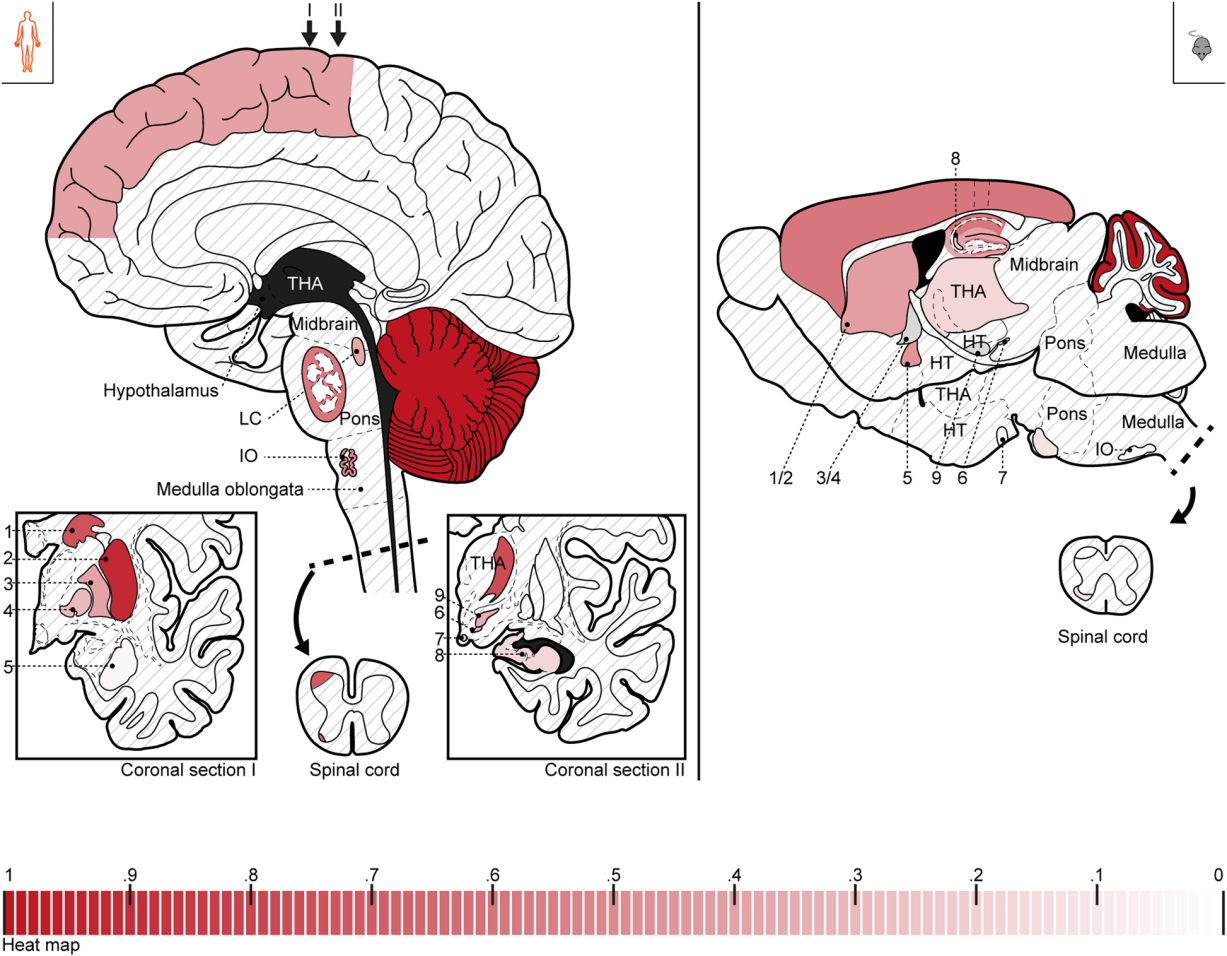
Heat map of SHANK2 expression in analyzed human and mouse brain regions (neuropil). The neuropil sum intensities (mean) of Additional file 4a,d are represented in the sketches, i.e., color intensities represent SHANK2 expression in the neuropil of each subregion. The applied heat map is shown at the bottom (intervals of 0.01, CB was dyed with the color intensity corresponding to 1). For the CTX and THA (mouse), the color from the analyzed subregion was extrapolated to the entire region since no strong differences were seen in the overview scans. The analyzed subregions are dashed. For the HC (mouse), intensities for all layers were determined by comparing the analyzed region(s) to the non-analyzed layers in overview scans. Overall, crossed out regions were not analyzed; gray regions were only analyzed in the other species. The sagittal sections are based on “Sobotta, Atlas der Anatomie; Kopf, Hals und Neuroanatomie” (24^th^ edition, p. 271) and the used mouse brain atlas (see “Methods”). The coronal sections are based on “The Atlas of the Human Brain.” 1 = CD, 2 = PUT, 3 = EGP, 4 = IGP, 5 = AMY (mouse: to present all regions, the extended AMY was colored as substitute), 6 = SN, 7 = HT, 8 = HC, 9 = STH (see Table S1)

Moderate neuropil sum intensities were measured in the CTX, AMY, and CPu (Additional file 4d and tilescan in Additional file 4c). Low expression was found in the HC, THA, pons, and in lamina I of the spinal cord (SC DH), which was recognizable as a striking immunoreactive band in overview scans. Sum intensities close to 0 were found for the HT, IO, and the SC VH, according to the largely missing puncta. In total, mean sum intensities of the analyzed regions ranged from 0 to 0.67. Overall, the relationship between regions was consistent with that observed for puncta density. The neuropil sum intensity data from human and mouse were used to create the heat maps in Fig. 2.

No strong variability was observed for the mean intensity of SHANK2 puncta in the analyzed brain regions (Additional file 4e).

As SHANK2 puncta density was much lower in some regions than in others, VGLUT1 was used as a marker for the majority of excitatory synapses to determine whether SHANK2 might not be present in all postsynapses but rather be expressed in certain subtypes. SHANK2 sum intensities were moderate in the murine CTX, AMY, and CPu and close to 0 in the HT and pons. In contrast, VGLUT1 was highly expressed in all these regions with no profound differences (Additional file 5a, c). Similarly, in a representative human brain (case 2), VGLUT1 densities in the AMY or ACC (regions with low SHANK2) were at average levels (Additional file 5b, d). Overall, these data strongly suggest that SHANK2 is absent from a subset of excitatory synapses (see SHANK2-/VGLUT1 + synapses marked with arrows in Fig. 4c).

### Characteristic SHANK2 expression in sublayers of analyzed brain regions

#### Hippocampus

**Fig. 3:**
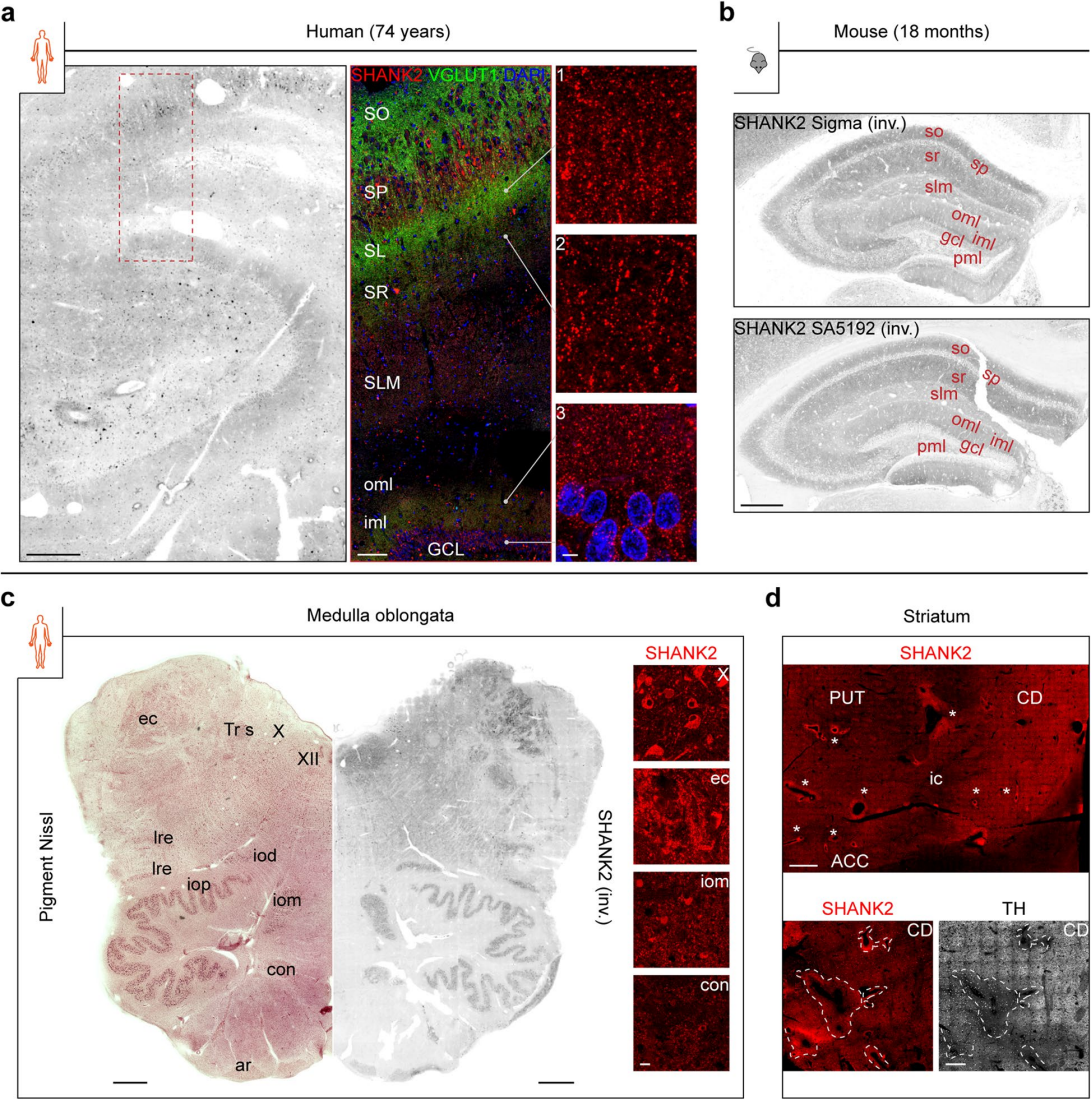
SHANK2 expression in the human hippocampus, medulla oblongata, and striatum. **a** SHANK2 staining (Sigma antibody) in a human hippocampus, representative for all cases. Representative stainings are shown as inverted (inv.) image (white = no signal). The dashed region is shown in the middle as merge image of SHANK2/VGLUT1/DAPI IF, high magnified insets of three layers are depicted on the right. Scale bars, 500 µm (left), 100 µm (middle), 5 µm (right). **b** SHANK2 distribution in a mouse hippocampus as revealed by two different antibodies. Staining is depicted in the same manner as in **a** (left). Scale bar, 300 µm. **c** SHANK2 IF (inv.) in a human medulla oblongata section (right), opposed to pigment Nissl staining (left). Scale bars, 1 mm. Insets of selected regions are shown on the right. Scale bar, 20 µm. **d** Vessel-related islands of enriched SHANK2 IF (*) in the human striatum. Scale bar, 1 mm (top). A CD section was stained for TH, de-stained, and re-stained for SHANK2 (bottom). Scale bar, 300 µm. ic = internal capsule

The sublayer-specific SHANK2 expression, besides the SL, was analyzed in both the human (Fig. 3a) and mouse (Fig. 3b) hippocampus, the central brain region for memory processing. In the human hippocampus, the highest IF levels were present in the SO, SP, and SL (inset 1, Fig. 3a) at the level of CA3 and the iml (inset 3, Fig. 3a) as confirmed by VGLUT1 co-staining (Fig. 3a, middle). SHANK2 puncta arranged like “beads-on-a-string” were observed in the SR (inset 2, Fig. 3a). Similar levels of SHANK2 IF were found for the pml; whereas the SLM and the oml showed weak SHANK2 expression. SHANK2 was present in the somata of granule cells and pyramidal neurons.

In the mouse hippocampus, the strongest SHANK2 expression was found in the *stratum oriens* (so), *stratum radiatum* (sr), and the inner molecular layer (iml). The outer molecular layer (oml) and the *stratum lacunosum et moleculare* (slm) showed weaker reactivity in a descending manner. SHANK2 intensity was lowest in the *stratum pyramidale* (sp), the granule cell layer (gcl), and the polymorph layer (pml). This pattern was observed using two different antibodies raised against the human (Sigma) and rat (SA5192) protein (Fig. 3b and Additional file 6g for intensity profile perpendicular to layer orientation).

#### Medulla oblongata

In addition to its expression in the principal subnucleus of the IO (iop), SHANK2 was found in the neuropil and in neurons of nearly all analyzed gray matter regions (Fig. 3c and Additional file 3d, right half and insets) including the dorsal accessory subnucleus (iod), the medial accessory subnucleus (iom), the conterminal (con) and arcuate nucleus (ar), the lateral reticular nucleus (lre), the nucleus of the spinal trigeminal tract (spV), the external cuneate nucleus (ec), the dorsal glossopharyngeal and vagal area (X), and the motor nucleus of the hypoglossal nerve (XII). However, SHANK2 was largely absent from the solitary tract (Tr s). These findings were confirmed by parallel pigment Nissl staining (Fig. 3c and Additional file 3d, left half).

#### Striatum

Tilescans revealed a striking pattern in the CD and the PUT, where moderately stained SHANK2 signals were interspersed with intensely stained structures around the presumable lumens of vessels (asterisks, Fig. 3d, top). Co-staining demonstrated weak VGLUT1 immunoreactivity (IR) in these regions compared to the surrounding area (data not shown). To investigate the relationship of these structures with matrix/striosome marker proteins, a tyrosine hydroxylase (TH)-stained CD section (Fig. 3d, bottom, right) was de-stained and re-stained for SHANK2 (Fig. 3d, bottom, left), a matrix-enriched protein present in axonal fibers from nigral neurons. Intriguingly, these islands exhibited weak TH IR, but not all TH-low areas showed a clear increase in SHANK2.

### Cell type-specific and subcellular expression of SHANK2 in humans and mice

**Fig. 4:**
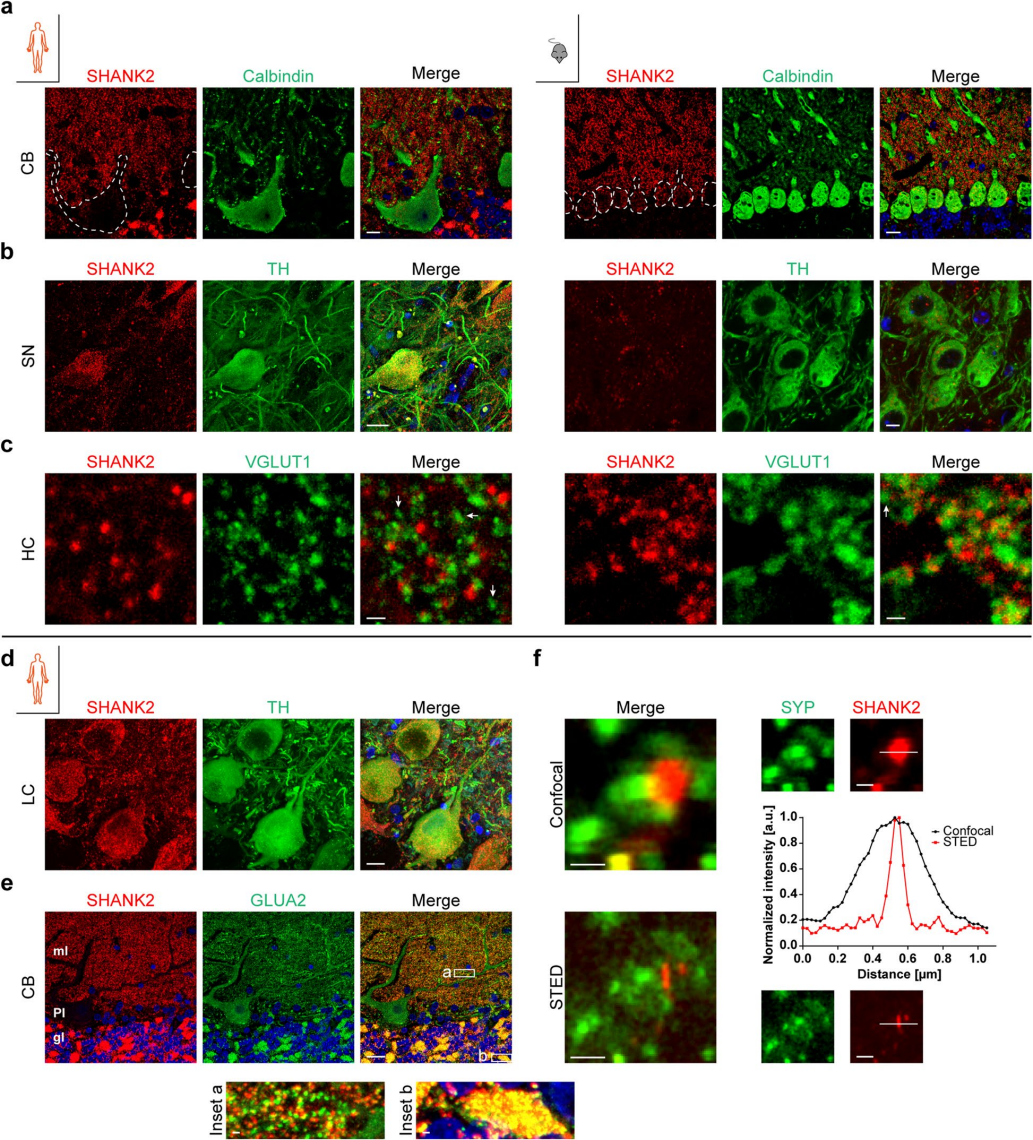
(Sub-)Cellular expression of SHANK2 in the human and mouse brain. **a**–**c** Staining of SHANK2 is opposed for human (left) and mouse (right) tissue sections. Co-staining of cellular (**a**,**b**) and synaptic (**c**) proteins in the CB, SN, and the SL of the HC. Arrows in **c** highlight SHANK2-/VGLUT1 + synapses. Scale bars, 10 µm (**a**), 20 µm (**b**, left), 5 µm (**b**, right), 1 µm (**c**). **d**, **e** Additional co-staining in the human LC (**d**) and CB (**e**). Scale bars, 15 µm (**d**), 20 µm (**e**), 1 µm (insets **e**). ml = molecular layer, Pl = Purkinje cell layer, gl = granular layer. DAPI (blue) is overlaid in **a**, **b** and **d**, **e**. **f** Confocal and STED microscopy of the same chemical synapse. Individual channels are shown as smaller images on the right, the graph shows the intensity profile across a SHANK2-positive PSD (white line) in confocal (black curve) and STED mode (red curve). Intensities were normalized to the maximum. Scale bars, 0.5 µm. a.u. = arbitrary unit

Initial experiments demonstrated the presence of somatic SHANK2 in the pyramidal neurons of the cerebral cortex (Fig. 1a). In co-staining experiments, SHANK2 IR was not observed in the soma of Purkinje cells (PCs) in the CB of either human (*n* = 4) or mouse (*n* = 3) specimens (Fig. 4a). In cerebellar sections from younger animals (P7, data not shown), SHANK2-positive puncta were enriched perisomatically, as previously described [37]. SHANK2 was expressed in TH-positive, catecholaminergic neurons of the SN (dopaminergic) in the human brain (Fig. 4b, left) and the LC (noradrenergic) (Fig. 4d), but in the mouse brain, TH-positive neurons exhibited low SHANK2 IR close to background level (lipofuscin) (Fig. 4b, right). Next, synaptic expression was confirmed by co-staining VGLUT1 in a human and mouse hippocampal section (SL, Fig. 4c), with both proteins found in close proximity. Due to the unique pattern in the human cerebellum (Fig. 4a), SHANK2 expression was further characterized by co-staining glutamate receptor subunit 2 (GLUA2) (Fig. 4e). In contrast to SHANK2, GLUA2 was detected in the soma of PCs and highly enriched along dendrites. Magnified insets from an apical dendrite in the molecular layer (ml) showed numerous SHANK2-/GLUA2-double-positive synapses (Fig. 4e, inset a). In the granular layer (gl), intense SHANK2- and GLUA2-positive puncta formed island-shaped structures, most likely representing cerebellar glomeruli (Fig. 4e, inset b) (in the mouse CB, SHANK2 was mainly found in the ml, see Fig. 4a). To further resolve the synaptic localization of SHANK2, super-resolution microscopy was performed with a stimulated emission depletion (STED) microscopy setup. Figure 4f shows a synapse in side view, which is characterized by its disk-shaped PSD and a distance to the presynaptic protein synaptophysin (SYP). Only STED microscopy could uncover the detailed structure, which was reflected by the full width at half maximum (FWHM) of an intensity line profile across a SHANK2-positive PSD, measuring 400 nm for the confocal mode and 84 nm for the STED mode.

### Conservation of SHANK2 isoforms in human and mouse brain regions

**Fig. 5:**
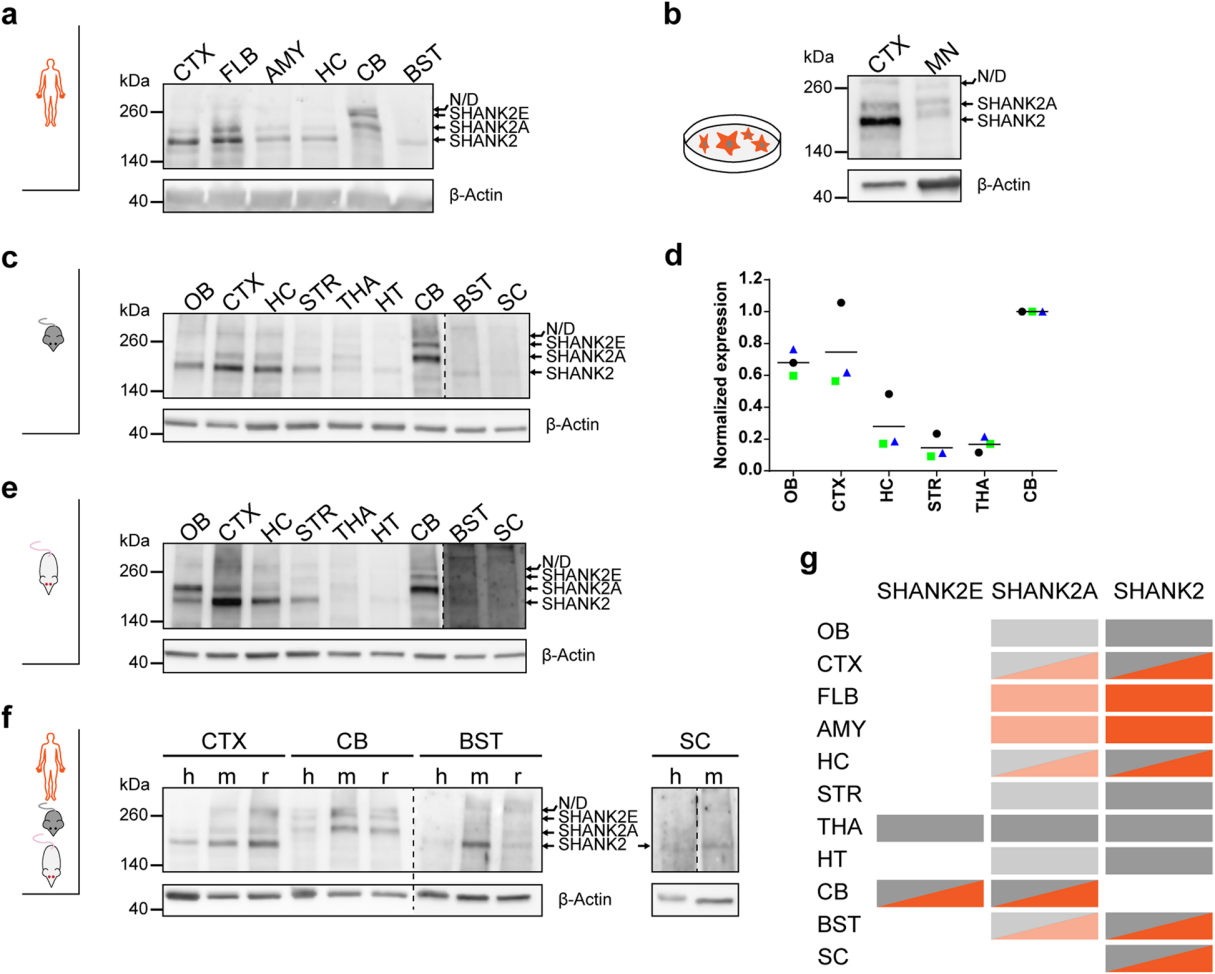
Conserved SHANK2 isoform expression in human and rodent brain regions. SHANK2 isoform expression in human (**a**), mouse (**c**), and rat (**e**) brain lysates. **b** SHANK2 isoforms in motor neuron (MN) lysates in comparison to a mouse CTX sample (here: 7 µg). **d** Quantification of total SHANK2E, SHANK2A, and SHANK2 in mouse brain regions, *n* = 3. Levels were normalized to the CB, horizontal lines indicate the mean. **f** The brain regions available from all species were blotted next to each other. Dashed lines indicate different exposure times for the left and right half of the membrane. **g** Summary of isoform expression in human (orange) and mouse (gray), shading represents expression strength. kDa = kilodalton, N/D = not defined, h = human, m = mouse, r = rat. The uncropped blots are shown in Additional file 9

The second major objective of this study was to evaluate the conservation of SHANK2 isoforms (as per nomenclature provided by references [9, 18]) between humans and rodents. To this end, brain lysates were analyzed using western blots. Firstly, the isoforms SHANK2A and SHANK2 were detected in the available human brain regions, such as the CTX, the frontal lobe (FLB), AMY, and the HC (Fig. 5a). In these regions, SHANK2 was expressed more strongly than SHANK2A. In the CB, similar levels of SHANK2A and SHANK2E were observed. In the brainstem (BST), SHANK2 was dominant, but longer exposure times indicated the presence of SHANK2A. In addition to the SC lysate, SHANK2 protein was present in MNs (d42) derived from human induced pluripotent stem cells (isoforms SHANK2 and SHANK2A, see also [38]) (Fig. 5b), thus confirming IF results. The lysates from mouse (Fig. 5c,d) and rat (Fig. 5e) CTX, CB, and BST showed the same pattern as the human lysates (for direct comparison, see Fig. 5f). The presence of isoform SHANK2A in the BST lysate was clearer for mice and rats than for humans (Fig. 5f, left). The lysates from the SC were difficult to separate using gel electrophoresis, but the presence of at least one SHANK2 isoform could be shown for human and mouse lysates (Fig. 5f, right). Secondly, all dissected mouse brain regions (Fig. 5c) were compared and the total expression of SHANK2 was quantified (Fig. 5d). As in the CTX lysate, SHANK2 was enriched compared to SHANK2A in the olfactory bulb (OB), striatum (STR), and HT. In the THA, all three isoforms were detected at similar levels (Fig. 5c). Total SHANK2 levels were highest in the CB, which was set to 1 for quantification (Fig. 5d). OB and CTX had similar levels (mean = 0.68 and 0.75, respectively). The HC showed moderate expression and the lowest levels were measured in the STR (mean = 0.15) and the THA (mean = 0.17). For qualitative assessments, the same regions were dissected from a rat brain (Fig. 5e), which largely agreed with the mouse data. A summary of the presence of the major isoforms in human (orange) and mouse (gray) brain regions is provided in Fig. 5g, where color intensities represent the expression levels.

### SHANK3 expression in the neuropil of core mouse brain regions

To our knowledge, only one semi-quantitative study has previously examined SHANK3 expression across multiple human brain regions [31]. Since our well-characterized homemade SHANK3 antiserum [18] was found unsuitable for intensity comparison in *post mortem* human tissue due to unclear background signals, it was only applied for IF staining in the mouse brain, a standard application.

Consistent with previous studies conducted in rodents [39, 40], the highest expression of SHANK3 was observed in the CPu (intensities from the other regions were normalized (0–1) to this region) and in the ACC as seen in the overview scans (Additional file 6a, b). Lower, but still high expression was detected in some hippocampal layers and the CTX. A detailed analysis of neuropil SHANK3 expression in core regions, similar to those included in the SHANK2 analysis, showed that puncta density was highest in the regions with the highest sum intensity, the CPu and the ACC, which were directly followed by the CTX (see Additional file 6c for representative images). The group with the second highest densities (AMY, THA, SC DH, and CB) followed the sum intensity data and the densities were between one and two thirds. Finally, the HC had only slightly higher puncta density than the regions with the lowest densities, which were the GP, HT, pons, and SC VH (densities between 0 and one third) (Additional file 6d).

CTX (M2), AMY, THA, and SC DH were the regions with the second highest SHANK3 sum intensities (Additional file 6e). These regions were closely followed by the CB; IR was also detected in the gl. Finally, among the analyzed regions/layers, the HC, GP, HT, pons, and SC VH had a SHANK3 expression close to 0. In total, SHANK3 sum intensities were mostly between 0 and 0.33, except for the CPu, the CTX, and the ACC. The mean intensities of SHANK3-positive puncta in the neuropil did not show large variations (Additional file 6f).

Finally, a direct comparison was made between SHANK2 and SHANK3 in the mouse brain, specifically in all analyzed regions and in the entire HC. Interestingly, intensity line profiles of SHANK2 and SHANK3 IF along the hippocampal layers revealed subtle differences for two layers: SHANK3 intensity in the pml was at least comparable to the iml; both regions have the second highest SHANK3 intensity (Additional file 6 g, top). This pattern largely coincided with VGLUT1 IF in mice [41], which is consistent with the role of SHANKs at excitatory synapses [1]. In contrast, SHANK2 IR in the pml was weak, but the iml was one of the three regions (so, sr, iml) with the highest SHANK2 intensities (Additional file 6 g, bottom). In the other regions, neuropil sum intensity levels were compared based on the relative expression compared to the region with the maximum intensity (CPu for SHANK3 and CB for SHANK2) (Additional file 6 h). The latter were also the regions with the most striking difference. Additionally, SHANK2 expression in the AMY was twice as high as SHANK3, while SHANK3 was twice as high in lamina I of the SC (always relative to the maximum intensity). Enrichment of SHANK3 in lamina I (Additional file 6a, inset) has been previously reported in a study that linked SHANK3 to pain processing [42]. Common to SHANK2 and SHANK3 proteins in mice was a strong expression in regions with high densities of excitatory synapses, such as the CTX and HC [43], and weak expression at synapses of the HT and the BST.

A comparison of SHANK3 protein expression between humans and mice is provided by the heat map in Additional file 7 (mouse data represents sum intensities of Additional file 6e). For human data, expression patterns were retrieved from the work of Wan et al. [31] and color-coded in two gradations: 100 and 50% (intensity described as “pale”, “light(er)”, or “weak” [31]).

### Conservation of SHANK3 isoforms in human and mouse brain regions

In an identical manner as for SHANK2, human, mouse, and rat brain regions were analyzed for the conservation of SHANK3 isoforms (major isoforms between 140 and 260 kDa). Owing to the plethora of currently known SHANK3 isoforms, a mouse PSD sample served as a reference for the human lysates (Additional file 8a). All major isoforms (SHANK3a, SHANK3c/d, and SHANK3e) were present in the human CTX, FLB, AMY (Additional file 8a), and HC (Additional file 8b), with SHANK3e appearing weaker in the FLB and HC sample. Notably, a shift towards the smaller isoforms was observed in the CB, BST, and SC. In the human CB, SHANK3c/d was enriched compared to SHANK3e, identical to the mouse (Additional file 8d) and rat lysates (Additional file 8e) (direct comparison: Additional file 8f). In the human BST, SHANK3e was the dominant isoform, while SHANK3c/d and SHANK3a were expressed at lower levels. Conversely, the rodent lysates showed the presence of SHANK3c/d and e isoforms, without detectable SHANK3a protein (Additional file 8f). In the rodent and human SC sample, SHANK3e was detected. While the human lysate showed the additional presence of SHANK3a, SHANK3c/d was found in the mouse lysate (Additional file 8f). In d42 MNs, all major SHANK3 isoforms were detected (see also [44]) when compared to a mouse CTX sample (Additional file 8c), which corresponded to the human cortex (Additional file 8f). Among the dissected mouse brain regions, an enrichment in smaller isoforms was observed in the HT, CB, BST, and the SC, similar to the human brain (Additional file 8d) (in line with CB in primer-specific analysis [45]). Of note, labeling of isoforms in line with previous studies was verified, e.g., by the enrichment of SHANK3a and SHANK3e in the STR (Additional file 8d and, e.g., [23, 45, 46]). Similar results were obtained with the rat lysates (Additional file 8e). A summary of the major isoforms’ presence in human (orange) and mouse (gray) brain regions is provided in Additional file 8g.

## Discussion

In this study, the expression of SHANK2 protein was analyzed for the first time in multiple human brain regions. Using high-resolution fluorescence microscopy and state-of-the-art image analysis tools, intensity and density of SHANK2-positive puncta in the neuropil were quantified in 19 brain regions of four elderly individuals. The advantage of 3D multiple labeling was exploited and cellular expression was compared in mouse and human brains. Strongest SHANK2 IF was detected in the mouse and human CB. Subtle differences were observed between mice and humans in some brain regions, but clear differences in the analyzed regions of the BST, AMY, and THA. To complement the existing data on SHANK3 expression in the brain, identical analysis was performed in the mouse brain, and subsequently, results were opposed to published human data [31]. Mice showed the highest levels of SHANK3 expression in the CPu, CTX, and distinct HC layers, which is consistent with human data [31]. SHANK2 and SHANK3 isoforms in brain lysates were largely conserved between mice and humans.

Despite the relevance of SHANKs in neurological disorders, protein expression has mainly been analyzed in rodent brains to date. So far, “The HPA” has been the only reference for SHANK2 expression in the human brain, but only basal ganglia, the CB, CTX, and HC have been annotated using semi-quantitative chromogenic labeling (3,3’-diaminobenzidine (DAB)). In contrast, in this study, a total of 19 brain regions was analyzed and cellular expression was studied using 3D multicolor IF labeling with the SHANK2 antibody from “The HPA.” This antibody was raised against a part of the human protein and belongs to the Prestige Antibodies (affinity purified with the antigen). Besides, it convinced with its specificity in staining and western blot experiments: In line with the images deposited in “The HPA,” the antibody visualized cellular and extracellular SHANK2 in the gray matter, while the white matter remained largely unlabeled except for interstitial neurons. It should be noted that qualitative labeling of postsynaptic proteins is challenging in *post mortem* human tissue and was not possible with several antibodies working in mouse tissue. While “The HPA” [35] reported unspecific bands in western blot applications of the SHANK2 antibody, we were able to detect with a similar dilution the typical SHANK2 isoforms at the correct size when compared to a well-characterized homemade SHANK2 antibody [10].

### SHANK2 synaptic expression

Age- and sex-matched human and mouse brain tissue were used to investigate the expression of SHANK2 protein and its conservation across species. The human CB exhibited the highest levels of SHANK2 IF, which is consistent with mouse brains. The relevance of SHANK2 in the human CB might be reflected by the observed motor delay and cerebellar dysfunction in ASD patients with *SHANK2* mutations [47]. Importantly, besides a role in motor function, the CB has also been ascribed a role in cognitive functions [48]. Moderate sum intensities of SHANK2 puncta in the neuropil were observed in all analyzed brain regions except for the AMY, ACC, HC, IGP, HT, SN (lower third), THA, and SC (upper third). First, this indicates that SHANK2 is expressed throughout the entire human brain. Second, in contrast to mouse brains, where BST regions and SC were largely devoid of SHANK2 (see also [22, 23]), SHANK2 was moderately respectively highly expressed in the human BST and SC. This implies a more widespread expression in humans compared to mice. Given that absolute SHANK2 puncta densities in the CB of humans and mice were comparable, the increased value for mice CTX compared to humans indicates a higher density of SHANK2-positive PSDs. This is in line with a previous EM study reporting up to ~ threefold higher synapse densities in mouse cortical areas when compared to human tissue [49]. Similarly, a proteomic study found enriched levels of SHANKs in mice cortical PSDs compared to human PSDs [29]. Yet, extended follow-up studies are required to resolve whether the observed intensity differences in cortical regions are due to sampling strategies or whether they persist after comparison of several cortical regions in humans and mice. For the THA, the neuropil density and sum intensity of SHANK2 puncta compared to CB levels were on average three times higher in humans than in mice. In contrast, puncta density and sum intensity in the AMY were considerably higher in mice compared to humans. Importantly, VGLUT1 staining of the AMY indicated a high density of excitatory synapses in humans, comparable to the striatum or the THA. Function-wise, enhanced anxiety in autistic individuals links the AMY to ASDs [50]. Consequently, studies showed structural changes in the AMY in ASDs [51, 52]. Interestingly, the analyzed lateral amygdaloid nucleus was histologically not affected in patients but similar to controls [50, 51]. On the contrary, SHANK3 is strongly expressed in the human lateral AMY [31] and caution must be taken with translating unique SHANK2 expression patterns to alterations in ASD patients, given the prevalence of *SHANK2* mutations in ASDs (~ 0.17% [47]). Interestingly, a recent transcriptomic study found one of the highest variabilities between mice and humans in the AMY and HT [27]. Overall, the here reported SHANK2 expression in the mouse brain was congruent with previous semi-quantitative studies [22, 23, 39]. However, to our knowledge, this is the first study presenting quantitative data of neuropil SHANK2 expression. The high density of VGLUT1 in regions with low SHANK2 puncta density, both in mice and humans, underscores the presence of different synaptic subtypes as indicated by a brain-wide study of PSD95-/SAP102-positive synapses [43].

Since only VGLUT1-positive synapses were analyzed, the following discussion on VGLUT1/VGLUT2 expression is aimed to highlight the relevance of VGLUT1 in the analyzed brain regions, also with respect to VGLUT3. In general, VGLUT1 is the dominant glutamate transporter and VGLUT2 is especially enriched in subcortical neurons [53, 54]. VGLUT1 and VGLUT2 show complementary expression in many brain regions, e.g., in the neocortex, thalamus, amygdala, hippocampus, and cerebellum of the rodent and human brain [54, 55]. In particular, cortical VGLUT1 IR is stronger than VGLUT2 IR in the analyzed layers III and V [54], while in humans, the pattern of both proteins is rather homogenous [55]. In the HC, where VGLUT1 dominates, VGLUT2 is enriched in the human CA3 [32] but low to absent in rodent SLu [54]. In the human AMY, VGLUT1 and VGLUT2 showed most pronounced IR in the basolateral part [55]; for rodents, IR was rather homogenous for both proteins [54]. Basal ganglia express considerable amounts of VGLUT1 and VGLUT2 [54, 55]. The rodent lateral THA expresses VGLUT1 and VGLUT2 [54]; in humans, only minor VGLUT2 IR was observed [55]. In rodents, the mammillary nuclei express more VGLUT1 than VGLUT2 [54]. In the brainstem, VGLUT2 dominates in the superior olive but strong VGLUT1 IR was described for the pontine nuclei (rodents, see [56]; analogous human regions not analyzed in [55]).

The observed inter-individual differences in synaptic densities appeared also in other studies among human “control” subjects (see, e.g., [57, 58]) and may be attributed to the style of living, as discussed in the context of varying severity of cognitive impairment in the presence of AD pathology [59]. The included individuals in this study had low AD stages and were representative for their age cohort [60]. In turn, the homogeneity observed among the analyzed mice might reflect the identical environment in which they grew up as well as their inbred status. From a methodological standpoint, it is important to note that this study specifically focused on neuropil SHANK2, i.e., synaptic SHANK2, rather than measuring mean intensity over an entire region, which would average various synaptic and cellular SHANK2 levels, different synaptic densities, and areas of white matter.

### Cellular and subcellular SHANK2 expression

A clear co-localization of SHANK2-/GLUA2-positive synapses was observed along proximal PC dendrites in the ml of the human CB. These synapses are presynaptically formed by climbing fibers from the inferior olivary nucleus, which may play a role in cognitive behavior [48]. Interestingly, climbing fibers in rat are VGLUT2-positive [54] and missing *VGLUT2* mRNA in the CB but IR in the ml suggest the same for the human CB [55]. Therefore, it is tempting to speculate that SHANK2 expression in humans is not restricted to VGLUT1-positive synapses. Detailed co-expression studies will reveal whether this holds true also for the HC, which would contrast with findings in mice [41]. In addition, SHANK2 expression at GLUA2-positive excitatory synapses in the entire ml suggests that parallel fibers arising from granule cells represent another presynaptic counterpart, the only other excitatory input in the ml. Intense SHANK2 IR was found exclusively in human cerebellar glomeruli of the gl, which most likely stems from PSDs of granule cells and/or Golgi cells. PCs were clearly devoid of SHANK2 IR (*n* = 4, three images per case), which is in line with mouse data from the present study and previous reports [20, 22]. This is an important finding given that initial studies reported on *Shank2* mRNA in PCs [40, 61] but subsequently, this detail (mRNA analysis) has been omitted. The discrepancy between mRNA (PC cell bodies and dendrites) [40] and protein expression (dendrites) is difficult to explain but may point towards a great importance of local translation [40]. Finally, the cases included in “The HPA” (age 19, 45, and 54 years) reflect the pattern described here in the three layers. Since comparable negative controls are not included in "The HPA", it cannot be ruled out that the “low” IR in the PCs is background IR.

In the HC, one striking difference is the pale IR in the sp of mice compared to the pronounced IR in the human brains. This is consistent, e.g., with VGLUT1 [32, 41], suggesting that in mice synaptic contacts along apical dendrites emerge in the SLu/SR, while in the human HC, excitatory contacts are clearly formed in the SP already.

Mostly striatal regions in the human brain were interspersed with islands with a rim of strong SHANK2 IF that was not observed in the mouse ACC and CPu. The pale center of these islands corresponded to the lumen of vessels. The areas with enriched SHANK2 IF may correspond to striosomes, which is consistent with low TH IF [62]. Since these SHANK2-high areas lacked VGLUT1 IF, it might be conceivable that they represent areas innervated by phospho-TH along vessels [63] or that the vessel-associated variability of staining simply reflects a fixation artifact due to high-pressure perfusion fixation. Overall, this emphasizes the necessity of large overview scans prior to in-depth analysis.

The expression analysis of large brain regions as shown here, e.g., for the human medulla oblongata, provides a basis for understanding shankopathies in humans. In particular, these data cannot be approximated with model organisms due to some unique features of the human BST: First, as shown for the arcuate nucleus and the nucleus pararaphales [24], some brain regions only exist in human brains. Second, the neurochemical properties of brain regions differ between species, e.g., as demonstrated for the medial vestibular nucleus and the nucleus prepositus hypoglossi [24]. Importantly, recent studies have reported alterations in BST regions in ASD patients and highlighted the importance of dissecting its precise role [64].

Except for Purkinje cells (see above), intense SHANK2-positive puncta were found in the soma of major neurons in all analyzed human brain regions including the immediately fixed human tissue resectate. In contrast, somatic signals in mice were often lower, even down to background levels (e.g., TH-positive cells). Of note, other somatic proteins could be detected with the staining protocol for mice (such as calbindin D28k and TH). The discrepancy between cellular levels of SHANK2 expression in mice and humans might be due to a general difference in SHANK expression, i.e., the midbrain seems to be largely devoid of IR in mice, or to different shuttling kinetics of somatic proteins.

### SHANK2 isoforms

The expression of SHANK2 isoforms was conserved among humans, mice, and rats and the pattern was consistent with mRNA data from humans [9]. However, unlike the findings of Leblond et al. [9], SHANK2E was not observed in all analyzed brain regions except for the CB, which could be related to region-specific splicing and translation or to the extremely low expression [9], below the sensitivity of immunoblots. In the CB, isoform SHANK2 was only detectable at the mRNA level [9]. Total SHANK2 levels in mice largely followed the dynamics of IF analysis. However, the different origins of data (analysis of neuropil puncta in IF versus lysates including additional compartments) and different percentages of white matter proteins per brain region (lysates) did not allow for reliable 1:1 comparison. Importantly, few studies have tackled isoform-specific functions, except for an overexpression study of SHANK2A [12]. It is assumed that only a certain percentage of existing isoforms have been identified so far. The presence of a small, fourth isoform, consisting only of the PDZ domain, was demonstrated at the mRNA level in the human brain [9].

### SHANK3

SHANK3 expression in the rodent brain has been intensively characterized, however mostly at the mRNA level and to a lesser degree at the protein level [39, 40, 61]. One semi-quantitative protein expression study in mice found the highest SHANK3 levels in the striatum, THA (both +++++), CA3 of the HC (++++), and the CTX (+++) [39]. Our data are consistent with these findings, except for the THA (~ 0.3 of striatal levels). Furthermore, low SHANK3 expression in the BST [39] and little *Shank3* mRNA in the rat HT [40] match the results from the present study. To date, SHANK3 expression in the human brain has been reported for the basal ganglia, CB, CTX, and HC in “The HPA” [65]. A more extensive study published in 2021 [31] analyzed SHANK3 expression in several brain regions. Owing to the nature of the data (DAB staining, no intensity analysis), direct comparison to our rodent data is only possible to a limited extent. High SHANK3 expression in the striatum, THA, HC, and CTX seems to be conserved between humans and mice. Other shared features are the considerably lower expression in the BST and HT and the obviously weaker SHANK3 expression in the GP in comparison to the CPu. Isoform expression between human, mouse, and rat lysates was found to be largely conserved. Minor differences in the BST and SC are most likely due to different anatomical conditions (BST, see also part on SHANK2) or technical aspects regarding protein separation in the SC lysate. In summary, strong SHANK IR was observed in several brain regions affected in ASD patients, such as the CTX, HC, CB, striatum, THA, and BST [48, 64, 66–69].

### Limitations

In the current study, SHANK expression was exclusively analyzed in aged male brains (71–89-years-old). A previous study on autism-associated genes did not report evidence for sex-specific expression patterns of *SHANK2* and *SHANK3* in the adult human CTX [70]. However, future studies including female brains may elucidate whether this finding is reflected at the protein level in additional brain regions and how SHANK2 levels behave during aging in both sexes.

## Conclusions

In summary, this is the first study to use quantitative IF to assess SHANK2 expression in multiple human brain regions. Despite the relevance of SHANK2 in ASDs, ID, and schizophrenia, most ground-lying studies on protein expression have been conducted in model organisms. In this study, we found overlapping expression patterns in humans and mice but we also revealed clear differences in specific regions. This highlights the irreplaceable role of *post mortem* human tissue in addition to studies in model organisms. The heat maps summarizing our results can serve as a reference for future brain-wide studies of SHANK expression in ASD patients, particularly in individuals with confirmed *SHANK* mutations. This could help to unravel how individual *SHANK* mutations, including those related to specific isoforms, lead to synaptic disturbances, providing a starting point for novel treatment approaches. It is also reasonable to assume that brain regions with high SHANK2 expression are more affected in case of mutation, which should be further investigated in future studies.

## Methods

### Human brain samples

*Post mortem* human brains were derived from permanent body donors of the Institute for Anatomy and Cell Biology, Ulm University (ethics committee of Ulm University). Details on the fixation protocol are included in [33]. Major inclusion criteria for the cases were low Alzheimer’s (AD) and Parkinson’s disease (PD) stages (Table 1). First, required tissue blocks were excised from the left hemisphere [exception: STH/THA block (case 2) and CD/PUT/EGP/IGP block (case 3 and 4)]. The Atlas of the Human Brain (from Mai, Majtanik, and Paxinos; 4^th^ edition) was used as a reference, the selected serial plate numbers (section levels) for all analyzed subregions are included in Table S1. Except for case 1 (cervical spinal cord), lumbar spinal cord sections were taken. Exemplary photos of sectioned tissue blocks are included in Additional file 2a-b. Second, tissue blocks were processed on a vibratome (Microm HM 650 V, Thermo Scientific, Walldorf) to obtain 100-µm-thick sections.

The epilepsy patient provided her written and informed consent for participation in the study. The study was approved by the ethics committee of Ulm University. Following surgery, the supplied part of the tissue resectate (origin: temporal pole) was immediately immersion-fixed in a solution according to Tutsch (anatomy course, for recipe: [33]) by the neuropathologist (estimated fixation delay ~ 45 min). After 24 h, the solution was changed to a 1% solution of formaldehyde. After 1 week, the tissue was cut with a vibratome (see above) and the CLARITY protocol was started.

### CLARITY

To improve staining quality and antibody penetration in formaldehyde-fixed human tissue, a modified CLARITY protocol was applied to the tissue sections as described previously [33].

### Immunofluorescence staining of human and mouse brain sections

IF staining after CLARITY was performed as described previously [33]. Primary antibodies are listed in Table S2, secondary antibodies in Table S3; for quantification in the *n* = 4 human brains, SHANK2 primary and rb-647 secondary antibodies were from the same LOT each. For STED microscopy, STAR RED anti-rabbit (Abberior Cat# STRED-1002-500UG, RRID:AB_2833015) and STAR ORANGE anti-guinea pig (STORANGE-1006-500UG, no RRID yet) (Abberior, Göttingen) were diluted 1:200. To avoid cross-reactivity, staining was finished for the rabbit primary antibody (incubation 24 h), before the guinea pig primary antibody (incubation 24 h) was added.

Available mouse brain and lumbar spinal cord sections (40 µm thick) from age-matched (18-month-old) male wildtype C57BL/6JRj mice (Janvier Labs, Le Genest-Saint-Isle, France) were stained following the protocol for human tissue, including primary and secondary antibody dilutions (exceptions: SHANK2 Sigma 1:80 for intensity quantification and secondary antibody incubation for 2 h). Heat-induced epitope retrieval was not performed since it lowered staining quality (data not shown). The figure number of the stained sections according to a mouse brain atlas (Franklin & Paxinos: The Mouse Brain, in stereotaxic coordinates; 3^rd^ edition) is indicated in Table S1.

Human and mouse tissue sections were finally mounted in ProLong Gold antifade (Invitrogen, Waltham, MA).

For Fig. 3d (bottom), first, TH was stained and acquired. Thereafter, the section and the secondary-only control were de-stained in clearing solution (for protocol see [33]). Control acquisitions with identical settings showed no residual signal. Next, SHANK2 staining was performed on the same sections. Abberior STAR dyes [STAR RED anti-rabbit, see above, and STAR RED anti-mouse (STRED-1001-500UG, no RRID yet)] and 97% TDE mounting medium (MMTDE-2001, Abberior) were used.

### Confocal microscopy

For intensity quantification, high-resolution *z*-stacks (2048 × 2048 pixels, zoom 1.5, step size = 0.22 µm) were acquired with the × 40 oil objective (ACS APO, numerical aperture (NA) 1.15, free working distance (WD) 270 µm) of a SPE confocal laser-scanning microscope (Leica, Wetzlar). Tilescans were acquired at 512 × 512 pixels using the LAS-X Navigator of the LAS-X software (Leica).

#### Human brains

For intensity quantification, first, a low-resolution tilescan spanning most of the subregion was performed to prevent acquisition in a non-representative area and to get an overview on how uniform the staining was per brain region. Second, three high-resolution (see above) images per subregion were acquired. Since in all cases, the CB had the highest intensity, the laser power (LP) was set in a way that SHANK2-positive puncta in the CB are shortly below saturation. Using this single LP and identical settings, all remaining subregions were acquired within the same day. This procedure was repeated for the other three cases.

#### Mouse brains

First, a low-resolution tilescan covering the entire tissue sections was run. In the first line, this helped to navigate through brain regions and second, as for the human brains, it provided an overview to select the ROIs for the high-resolution images. In total, two images were acquired, one per hemisphere. All mouse brain sections were acquired on the same day, using the same strategy as described above for the human samples.

### STED microscopy

Images were acquired on a STEDYCON system (Abberior, Göttingen) connected to an Axio Imager.Z1 microscope (Zeiss, Oberkochen) using an α Plan-Apochromat × 100/1.46 Oil DIC (UV) VIS-IR objective. Excitation lasers at 640 nm (STAR RED, dye 1) and 561 nm (STAR ORANGE, dye 2) were used, the STED laser had a wavelength of 775 nm (both dyes) and was set to 50% (dye 1) and 88% (dye 2), which corresponds to an equal resolution. Pixel dwell times were 5 µs (confocal) and 10 µs (STED). Line accumulations were activated and the pixel size for confocal and STED mode was 25 nm.

The intensity profile was generated in Fiji (ImageJ, NIH, Bethesda, MD) (“plot profile” function), and the values were normalized to the maximum intensity. The final graphs were set up in GraphPad Prism (GraphPad Software, La Jolla, CA). The graphs were generated with raw data; only for visualization, a Gaussian blur with *σ* = 1.0 was applied to the images and contrast was adjusted in Fiji. The FWHM of the Gaussian-fitted graphs was determined in OriginPro (OriginLab, Northampton, MA) software.

### (Pigment) Nissl staining of tissue sections

Pigment (lipofuscin) Nissl staining of 100-µm-thick free-floating human tissue sections was performed as described previously [32]. Forty-µm-thick mouse tissue sections were rinsed in Millipore water and stained in an aqueous solution of 0.1% cresyl violet (acetate) (Merck, Darmstadt) for 2 min. Thereafter, staining was differentiated in 96% ethanol supplemented by a few drops of 10% acetic acid (based on https://pathologycenter.jp/method-e/nissl.html). Finally, tissue sections were dehydrated in an increasing alcohol series and mounted in Permount (Fisher Scientific).

Overview scans of human and mouse (pigment) Nissl staining were acquired using the × 4 dry objective (CFl Plan Apo λ, NA 0.2, WD 20 mm) of a Biorevo BZ-X810 microscope (Keyence, Neu-Isenburg) as described elsewhere [32]. To correct for unevenness in some field of views in the mouse sections, tiles were acquired as *z*-stack and the full focus projection is shown.

### Human and rodent brain lysates

Human brain lysates were purchased from Clontech/Takara (Kusatsu, Japan): Human Brain, Amygdala Protein Medley (Cat# 635317), Human Brainstem Protein Medley (Cat# 635325), Human Brain, Cerebellum Protein Medley (Cat# 635326), Human Brain, Cerebral Cortex Protein Medley (Cat# 635323), Human Brain, Frontal Lobe Protein Medley (Cat# 635318), Human Brain, Hippocampus Protein Medley (Cat# 635319), Human Spinal Cord Protein Medley (Cat# 635324).

Mouse brain and spinal cord (entire spinal cord) lysates were derived from male 6-month-old wildtype C57BL/6JRj mice, and rat lysates were from female Sprague Dawley rats (both Janvier Labs) (license o.103). Dissected brain regions were homogenized on ice in buffer 1 and centrifuged to obtain the S1 fraction (for detailed protocol, see [71]). The protein concentration of the supernatant (S1) was determined by the Bradford assay. The PSD sample from a mouse brain (Additional file 8a) was obtained following the subcellular fractionation protocol in [71] (P3 fraction), and 0.5 µl was applied for western blotting.

### Motor neuron lysates

Generation of human induced pluripotent stem cells and differentiation into motor neurons (MNs) (control) was performed as described previously [44]. MN lysates were generated following the protocol in [44]. The protein concentration was determined by the Bradford assay.

### Recombinant proteins

COS7 cells were transfected with GFP-Myc-*SHANK3a* [72] and mCherry-DEST-*SHANK2* wildtype [73] constructs (full-length human sequences), a GFP-*Shank1* construct (rat sequence [3]), and the corresponding empty vectors. Cells were harvested on ice in lysis buffer (µMACS GFP Isolation Kit, Miltenyi Biotec, Bergisch Gladbach) containing protease inhibitor cocktail (Roche, Mannheim). Lysates were sonicated and left on ice for 30 min. Following centrifugation at 10,000 × *g* for 10 min (4 °C), the protein concentration (supernatant) was measured with the Bradford assay. 15 µg per construct was prepared for western blotting.

### Western blots

Human Protein Medley was prepared for western blotting according to the manufacturer’s protocol (PT1602-1). For detection of SHANK2, 50 µg of each human lysate and 14 µg of each mouse or rat lysate were prepared. For SHANK3 detection, 30 µg of each human lysate, 7 µg of each mouse lysate, and 5 µg of each rat lysate were prepared. For the MN lysates, 30 µg was prepared. Samples and a protein ladder (Spectra Multicolor Broad Range, Thermo Scientific) were separated in 4–15% TGX precast gels (Bio-Rad, Munich). Western blotting was performed using the Trans-Blot Turbo Transfer system (Bio-Rad). Blocking was performed in 5% milk (Honeywell Fluka, Steinheim) plus 0.2% Tween20 (Carl Roth, Karlsruhe) for 2 h. Antibodies were diluted in blocking solution, dilutions for primary antibodies are included in Table S2 and those for secondary antibodies (incubation: 2 h) in Table S3. Signals were visualized using the Clarity Western ECL Substrate kit (Bio-Rad) and finally recorded at a MicroChemi 4.2 station (DNR Bio-Imaging Systems, Neve Yamin, Israel).

To increase the sensitivity of the specificity blot in Additional file 1c, the membrane with the SHANK1 and SHANK3 constructs was incubated separately with the SHANK2 antibody to prevent absorption of most antibodies by the SHANK2 construct.

### Analysis of immunofluorescence staining

#### Deconvolution

Prior to each analysis, confocal raw images were deconvolved using Huygens Essential (Scientific Volume Imaging, Hilversum, The Netherlands) software. Intensity preservation after deconvolution with the maximum likelihood estimation (MLE) had been demonstrated in a white paper issued by SVI (https://svi.nl/IntensityPreservation). First, following the establishment of the parameters in the Deconvolution Wizard, templates for microscopic and deconvolution parameters were created and loaded in the Workflow Processor. A fix, absolute background value was determined by calculating the mean background of the first, middle, and last acquired image per experiment. The signal to noise ratio (SNR) was set to 15 for SHANK2 intensity quantification (SNR = 10 for mouse staining).

#### Intensity and density measurements

Deconvolved images were analyzed in Imaris software (Oxford Instruments, Abingdon, UK). For the main analysis shown in Fig. 1b,c, Additional file 4a-b,d-e, and Additional file 6d-f, the “surface” creation wizard was used with identical parameters (human and mouse, each). In detail, to account for intensity differences between regions, the “background subtraction (local contrast)” option was selected (with fix thresholds for all brain regions). Surfaces smaller than ten voxels were excluded. In total, three (mouse: four) equal sized ROIs per image were analyzed. The DAPI channel was constantly overlaid to ensure analysis outside of cell somata. For the final graphs, first the mean (for parameter mean intensity) or sum (for parameters sum intensity and puncta density) of all detected surfaces per image was calculated. Second, the mean of all three (mouse: two) images per subregion was calculated and normalized to the region with the highest intensity (CB for SHANK2, CPu for SHANK3) as performed in a similar study [43]. These normalized values (0–1) are plotted per case in Fig. 1b,c, Additional file 4a-b,d-e, and Additional file 6d-f together with the mean for all cases (horizontal line).

For Additional file 1d, layer III and V were excised from tilescan acquisitions spanning the entire cerebral cortex. The SNR was set to 10 (Huygens) and SHANK2-positive puncta were detected using the “surface” creation wizard (Imaris). Three ROIs per layer were analyzed and their mean was normalized to a reference case, which was the same per experiment (case 2).

For VGLUT1 analysis (Additional file 5c-d), following deconvolution, positive puncta were detected using the “surface” creation wizard (Imaris). For number of images and ROIs, see SHANK analysis. Owing to a clearly observable decrease in intensity throughout the *z*-stack, only the upper *z*-plane was considered for analysis (human: full *z*-range: 1.98 µm). All brain regions (mouse and human) were acquired within 1 day for each species.

#### SHANK2 / MAP2

First, following deconvolution (SHANK2 channel), SHANK2-positive puncta were detected with the “spot” creation wizard (Imaris) and dendrites were outlined with the “surface” creation wizard (both with identical parameters for all cases). Second, the number of spots within a maximum distance of 1.6 µm to the surface of a dendrite was filtered. This value corresponds to the mean spine length of apical and basal dendrites (anterior cingular gyri) of a 85-year-old human according to [34]. To calculate the spots number per µm dendrite, the dendrite length was measured using the “filament” creation wizard. In total, five images per case were acquired (*z* = 8.13 µm, step size = 0.22 µm) and a total of ten dendrites per case was analyzed. For case 1, most dendrites were orientated perpendicular to the focal plane, which is why this case was not included.

#### Western blot analysis

Intensities (Fig. 5d) were quantified in Image Lab 6.1 software (Bio-Rad). Western blots from *n* = 3 mice were analyzed. The background-corrected volume (sum intensity) of SHANK2E, SHANK2A, and SHANK2 bands was normalized to the background-corrected volume of the β-actin signal. For comparison, raw values were normalized to the region with the highest expression of SHANK2 (CB). Individual mice and the overall mean are presented in the graph. BST and SC were excluded from quantification since SHANK2 detection in these lysates required longer exposure times.

In all figures, non-deconvolved brightness-/contrast-adjusted (Imaris or Fiji) images are shown if not stated differently (*z*-stacks as maximum intensity projection). All graphs were set up in GraphPad Prism software and final figures were created in Adobe Illustrator (Adobe, San José, CA).

### Supplementary Information

**Additional file 1.** Specificity and quality tests with the SHANK2 Sigma antibody used for immunostaining. **a** Antigenic regions of the SHANK2 Sigma (red) and the homemade antibody (brown) used in the present study in the three isoforms. Isoforms were drawn according to [18], sketches are not true to scale. ANK = ankyrin repeats, SH3 = Src homology 3, PDZ = PSD-95/Discs large/zonula occludens-1, PRC = proline-rich clusters, SAM = sterile alpha motif. **b** SHANK2 staining in the temporal CTX of a 21-years (yrs)-old female (f). DAPI is depicted in blue. Scale bar, 5 µm. **c** Western blot showing a specific reaction of the SHANK2 Sigma antibody with its target protein, but not with SHANK1 or SHANK3; empty vectors were also included. The membrane was cut along the red dashed lines and incubated with the indicated antibodies (left). The left part of the membrane (dashed blue line; until 50 kDa band) was divided after signal detection, the left side was incubated with a SHANK1 antibody and the right side with a SHANK3 antibody (right). **d** SHANK2 density in CTX layers III and V (SFG) of cases 1-4. Case 2 was part of all experiments and served as a reference to which the other cases were normalized to. Absolute values of case 2 per experiment deviated by a maximum of 19%. Scale bar, 2 µm. **e** SHANK2 puncta per µm of MAP2-positive dendrite, cases 2-4 (SFG). Deconvolved image, scale bar, 2 µm. **f** Comparison of SHANK2 isoforms in the human frontal lobe lysate with the two antibodies introduced in **a**. **g** Isoforms in the human (hum) CTX in comparison to mouse (ms) and rat lysates (various amounts) as revealed by the SHANK2 Sigma antibody. **h** Multiple sequence alignment (created with Clustal Omega, version 1.2.4, EMBL-EBI) of the SHANK2 antigen (APREST71536, Sigma, human (hum) sequence, 130 amino acids (AAs)), the corresponding mouse (ms), and rat sequence (UniProt IDs are indicated). Non-conserved AAs are highlighted in yellow. “Weakly similar properties” (.), “strongly similar properties” (:), and conserved AAs (*) were annotated by the alignment tool (see [74]). kDa = kilodalton, N/D = not defined. The uncropped blots are shown in Additional file 10

**Additional file 2.** Negative controls for IF staining of human tissue. **a** Exemplary photos of dissected human brain regions. **b** Identification of subregions in the human AMY (left) by pigment Nissl staining (right). Dashed lines indicate the borders of the lateral (lat) and basolateral (basolat) nuclei. Scale bar, 1 mm. **c** Exemplary images of secondary antibody (ab) and IgG controls of five regions; images for each control were acquired and processed with identical settings. Scale bars, 100 µm, inset CB, 1 µm. ml = molecular layer, Pl = Purkinje cell layer, gl = granular layer. **d** Intracellular (I.c.) SHANK2 and lipofuscin can be distinguished by their shape, intensity, and distribution (exemplary image of the THA). Scale bar, 5 µm. **e** SHANK2 staining and secondary ab control (ctrl), acquired in high-resolution with identical settings, image processing identical. Scale bar, 5 µm. Lf = lipofuscin

**Additional file 3.** SHANK2 signal along neurites and somatic SHANK2 in the pallidum, SN, and LC. **a**-**c** SHANK2 (left) and MAP2 (middle) co-staining is shown for the single channels and as merge image (right) for cases 1 (**a**), 2 (**b**), and 4 (**c**). A zoom-in from the SHANK2 and merge image (white dashed region) is provided on the outer right side. Images represent maximum intensity projections with z = 5.94 µm. Scale bars, 20 µm (left), 5 µm (zoom-in right). **d** SHANK2 distribution in a medulla oblongata section from a second case; slightly different cutting level than in Fig. 3c. Nissl staining is shown on the left side, inverted (inv.) SHANK2 IF on the right side. Scale bars, 1 mm. **e** Inverted (inv.) SHANK2 IF in the SN and LC shows somatic SHANK2 and SHANK2 aligned along neurites (red arrows). Scale bars, 30 µm (SN), 20 µm (LC)

**Additional file 4.** Sum and mean intensity of SHANK2-positive puncta in the neuropil of human brain regions. **a**, **b** Graphs are based on the analysis in Fig. 1b; the normalized sum intensity (**a**) and mean intensity (**b**) of SHANK2-positive puncta in the neuropil is plotted (*n* = 4). **c** Nissl staining and overview scan of SHANK2 IF in a coronal mouse section. The figure number refers to the used brain atlas (see “Methods”). Scale bars, 500 µm. **d**, **e** Graphs are based on the analysis in Fig. 1c; (*n* = 3), identical to humans in **a**, **b**. In **a**, **b** and **d**, **e**, values were normalized to the CB; means of the analyzed *n* are indicated by horizontal lines

**Additional file 5.** Synaptic density in human and mouse brain regions as determined by VGLUT1. **a**, **b** VGLUT1 IF in most of the mouse (**a**) and human (**b**) brain regions included in the SHANK2 analysis (*n* = 1 each). EGP and IGP were excluded since VGLUT1 protein is not localized in these regions of the human brain [55]. Scale bars, 5 µm. **c**, **d** VGLUT1 puncta density for mouse (2D) (**c**) and human (3D) (**d**). Owing to an obvious decrease in VGLUT1 intensity from the superficial to lower z-planes in mouse, analysis following deconvolution was performed only in the superficial layer (2D)

**Additional file 6.** SHANK3 expression in core mouse brain regions. **a** Acquisition strategy: Low-resolution overview scans of each coronal mouse section were performed prior to selecting the positions for the high-resolution images (white rectangles with cross). The figure number refers to the used brain atlas (see “Methods”). The small inset in the left image shows the enrichment of SHANK3 in the SC DH lamina I; the dashed line indicates the section border. Scale bar, 50 µm. **b** Nissl staining and overview scan of SHANK3 IF in a coronal mouse section. Scale bars, 500 µm. **c** Representative images of mouse SHANK3 protein expression in all analyzed subregions. Brightness/contrast adjustments were performed identically for all regions. Scale bar, 5 µm. Analysis of SHANK3-positive puncta density (**d**), their sum intensity (**e**), and their mean intensity (**f**) is displayed as described for SHANK2 in Fig. 1c (*n* = 3). For SHANK3 analysis, ACC and GP from two mice were additionally included. In **d**-**f**, values were normalized to the CPu; means of the analyzed *n* are indicated by horizontal lines. **g** SHANK3 and SHANK2 intensity in the mouse HC. Intensity profiles were generated on raw data (white boxes, 90° rotated). Intensities (int.) were normalized to the maximum values. Scale bars, 500 µm. a.u. = arbitrary unit. **h** Comparison of SHANK2 and SHANK3 intensity, means were combined from Additional file 4d (SHANK2, dashed line) and Additional file 6e (SHANK3, continuous line)

**Additional file 7.** Heat map of SHANK3 expression in human and analyzed mouse brain regions (neuropil). The sum intensities (mean) of Additional file 6e are represented in the mouse sketches, i.e., color intensities represent SHANK3 expression in the neuropil of each subregion. The applied heat map is shown at the bottom (intervals of 0.01, CPu was dyed with the color intensity corresponding to 1). Information for the human brain was retrieved from [31]. Regions described to stain “pale”, “light(er)”, or “weak” were dyed with the color for 0.5, regions with higher SHANK3 protein expression with the color intensity corresponding to 1. For the CTX and THA (mouse), the color from the analyzed subregion was extrapolated to the entire region since no strong differences were seen in the overview scans. The analyzed subregions are dashed. For the HC (mouse), intensities for all layers were determined by comparing the analyzed region(s) to the non-analyzed layers in overview scans. Overall, crossed out regions were not analyzed; gray regions were only analyzed in the other species. The sagittal sections are based on "Sobotta, Atlas der Anatomie; Kopf, Hals und Neuroanatomie" (24^th^ edition, p. 271) and the used mouse brain atlas (see “Methods”). The coronal sections are based on "The Atlas of the Human Brain". 1 = CD, 2 = PUT, 3 = EGP, 4 = IGP, 5 = AMY (mouse: to present all regions, the extended AMY was colored as substitute), 6 = SN, 7 = HT, 8 = HC, 9 = ACC (see Table S1)

**Additional file 8.** Conserved SHANK3 isoform expression in human and rodent brain regions. SHANK3 isoform expression in human (**a**), mouse (**d**), and rat (**e**) brain lysates (for **d**, **e**: 8% gels). The PSD sample in **a** was derived from a mouse brain. **b** SHANK3 isoforms in human CTX and HC lysates (here: 25 µg each, separated in 8% gels), which were used for initial experiments. **c** SHANK3 isoforms in motor neuron (MN) lysates in comparison to a mouse CTX sample. **f** The brain regions available from all species were blotted next to each other. Dashed lines indicate different exposure times for the left and right half of the membrane. **g** Summary of isoform expression in human (orange) and mouse (gray), shading represents expression strength. kDa = kilodalton, unlabeled arrow = 300 kDa band, h = human, m = mouse, r = rat. The uncropped blots are shown in Additional files 11 and 12

**Additional file 9.** Raw data for western blots. Uncropped membranes are included as raw data. Membrane cutting before primary antibody incubation is indicated by red lines and those parts of the membrane that are shown in a figure are labeled with the respective antibody. If not the whole part is shown, the numbers of the extracted lanes are highlighted in red. Panel labeling in Additional files 9, 10, 11, and 12 refers to the original figures; the assignment is as follows: Figure 5 → Additional file 9. M = marker

**Additional file 10.** Raw data for western blots. Uncropped membranes are included as raw data. Membrane cutting before primary antibody incubation is indicated by red lines and those parts of the membrane that are shown in a figure are labeled with the respective antibody. If not the whole part is shown, the numbers of the extracted lanes are highlighted in red. Panel labeling in Additional files 9, 10, 11, and 12 refers to the original figures; the assignment is as follows: Additional file 1 → Additional file 10. M = marker

**Additional file 11.** Raw data for western blots. Uncropped membranes are included as raw data. Membrane cutting before primary antibody incubation is indicated by red lines and those parts of the membrane that are shown in a figure are labeled with the respective antibody. If not the whole part is shown, the numbers of the extracted lanes are highlighted in red. Panel labeling in Additional files 9, 10, 11, and 12 refers to the original figures; the assignment is as follows: Additional file 8 → Additional files 11 and 12. M = marker

**Additional file 12.** Raw data for western blots. Uncropped membranes are included as raw data. Membrane cutting before primary antibody incubation is indicated by red lines and those parts of the membrane that are shown in a figure are labeled with the respective antibody. If not the whole part is shown, the numbers of the extracted lanes are highlighted in red. Panel labeling in Additional files 9, 10, 11, and 12 refers to the original figures; the assignment is as follows: Additional file 8 → Additional files 11 and 12. M = marker

**Additional file 13: Table S1.** Analyzed regions (SHANK2) in human and mouse brains. **Table S2.** Primary antibodies used for IF staining and western blots. **Table S3.** Secondary antibodies used for IF staining (not STED) and western blots

## Data Availability

All data generated or analyzed during this study are included in this published article and its supplementary information files. Data not included into the manuscript may be released upon reasonable request to the corresponding author and in compliance with the ethics votes.

## Abbreviations

3D: Three-dimensional
ACC: Accumbens nucleus
AD: Alzheimer’s disease
AMY: Amygdala
ANK: Ankyrin repeats domain
ar: Arcuate nucleus
ASD: Autism spectrum disorder
BST: Brainstem
CB: Cerebellum
CD: Caudate nucleus
con: Conterminal nucleus
DAB: 3,3’-Diaminobenzidine
DH: Dorsal horn
ec: External cuneate nucleus
EGP: External segment of the globus pallidus
FLB: Frontal lobe
FWHM: Full width at half maximum
gcl: Granule cell layer
gl: Granular layer
GLUA2: Glutamate receptor subunit 2
HC: Hippocampus
HPA: The Human Protein Atlas
HT: Hypothalamus
ID: Intellectual disability
IF: Immunofluorescence
IGP: Internal segment of the globus pallidus
iml: Inner molecular layer
IO: Inferior olive
iod: Dorsal accessory subnucleus
iom: Medial accessory subnucleus
iop: Principal subnucleus of the IO
IR: Immunoreactivity
LC: Locus coeruleus
LP: Laser power
lre: Lateral reticular nucleus
ml: Molecular layer
MLE: Maximum likelihood estimation
MN: Motor neuron
OB: Olfactory bulb
oml: Outer molecular layer
PC: Purkinje cell
PD: Parkinson’s disease
PDZ: PSD-95/Discs large/zonula occludens-1
PMDS: Phelan-McDermid syndrome
pml: Polymorph layer
PRC: Proline-rich clusters
PSD: Postsynaptic density
PUT: Putamen
SAM: Sterile alpha motif
SC: Spinal cord
SFG: Superior frontal gyrus
SH3: Src homology 3
SL: *Stratum lucidum*
slm: *Stratum lacunosum et moleculare*
SN: Substantia nigra, pars compacta
SNR: Signal to noise ratio
so: *Stratum oriens*
sp: *Stratum pyramidale*
spV: Nucleus of the spinal trigeminal tract
sr: *Stratum radiatum*
STED: Stimulated emission depletion
STH: Subthalamic nucleus
STR: Striatum
SYP: Synaptophysin
TH: Tyrosine hydroxylase
THA: Thalamus
Tr s: Solitary tract
VGLUT1: Vesicular glutamate transporter 1
VH: Ventral horn
WD: Working distance
