# Supplementary figures and images for: Expression profiles of the autism-related SHANK proteins in the human brain
Source: BMC Biol. 2023 Nov 13;21:254. doi: 10.1186/s12915-023-01712-0 (PMC10641957; doi:10.1186/s12915-023-01712-0)

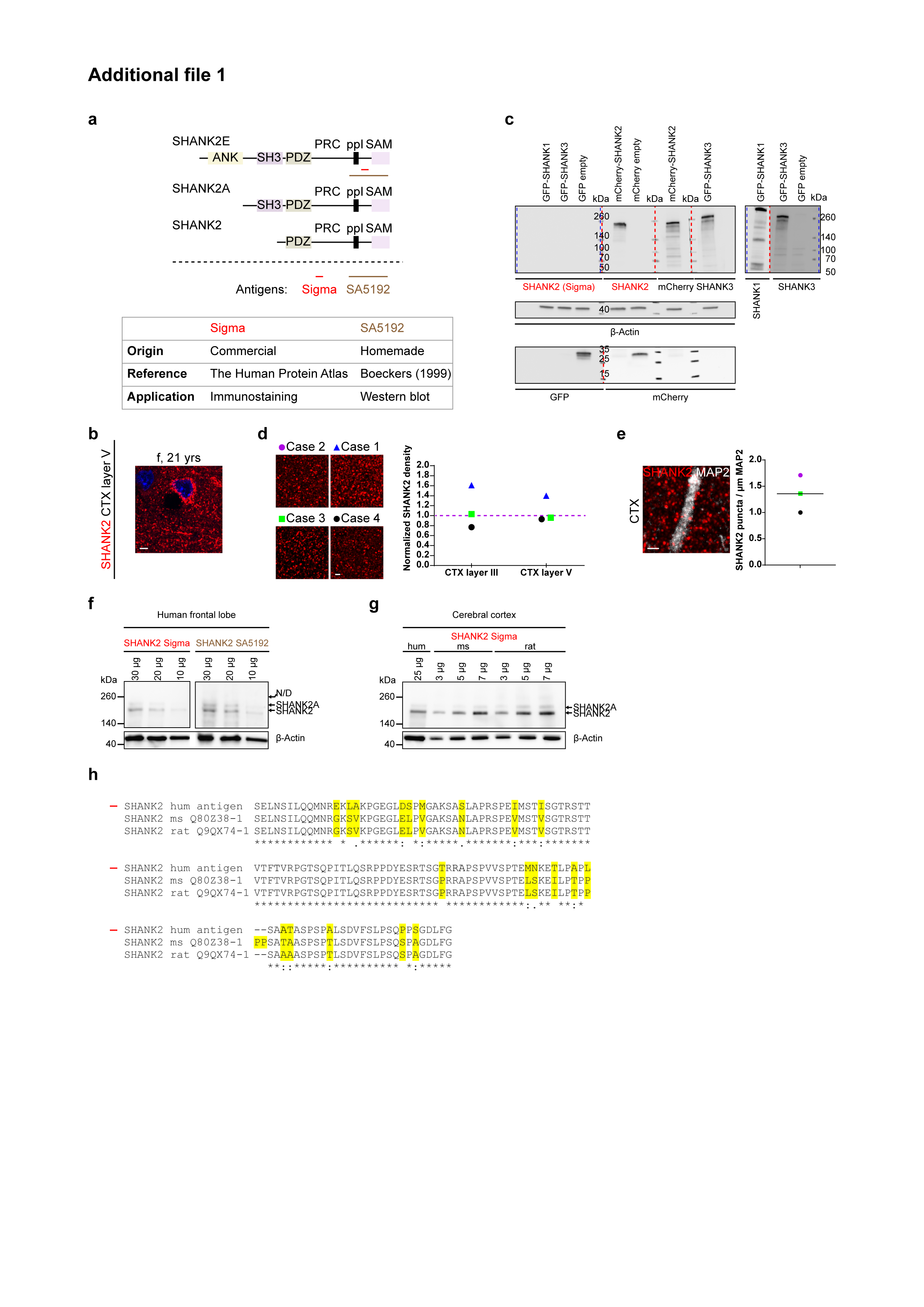

Supplement: Supplementary file 1 — Additional file 1. Specificity and quality tests with the SHANK2 Sigma antibody used for immunostaining. a Antigenic regions of the SHANK2 Sigma (red) and the homemade antibody (brown) used in the present study in the three isoforms. Isoforms were drawn according to [18], sketches are not true to scale. ANK = ankyrin repeats, SH3 = Src homology 3, PDZ = PSD-95/Discs large/zonula occludens-1, PRC = proline-rich clusters, SAM = sterile alpha motif. b SHANK2 staining in the temporal CTX of a 21-years (yrs)-old female (f). DAPI is depicted in blue. Scale bar, 5 µm. c Western blot showing a specific reaction of the SHANK2 Sigma antibody with its target protein, but not with SHANK1 or SHANK3; empty vectors were also included. The membrane was cut along the red dashed lines and incubated with the indicated antibodies (left). The left part of the membrane (dashed blue line; until 50 kDa band) was divided after signal detection, the left side was incubated with a SHANK1 antibody and the right side with a SHANK3 antibody (right). d SHANK2 density in CTX layers III and V (SFG) of cases 1-4. Case 2 was part of all experiments and served as a reference to which the other cases were normalized to. Absolute values of case 2 per experiment deviated by a maximum of 19%. Scale bar, 2 µm. e SHANK2 puncta per µm of MAP2-positive dendrite, cases 2-4 (SFG). Deconvolved image, scale bar, 2 µm. f Comparison of SHANK2 isoforms in the human frontal lobe lysate with the two antibodies introduced in a. g Isoforms in the human (hum) CTX in comparison to mouse (ms) and rat lysates (various amounts) as revealed by the SHANK2 Sigma antibody. h Multiple sequence alignment (created with Clustal Omega, version 1.2.4, EMBL-EBI) of the SHANK2 antigen (APREST71536, Sigma, human (hum) sequence, 130 amino acids (AAs)), the corresponding mouse (ms), and rat sequence (UniProt IDs are indicated). Non-conserved AAs are highlighted in yellow. “Weakly similar properties” (.), “strongly similar properties” (:), [file 12915_2023_1712_MOESM1_ESM.tif]

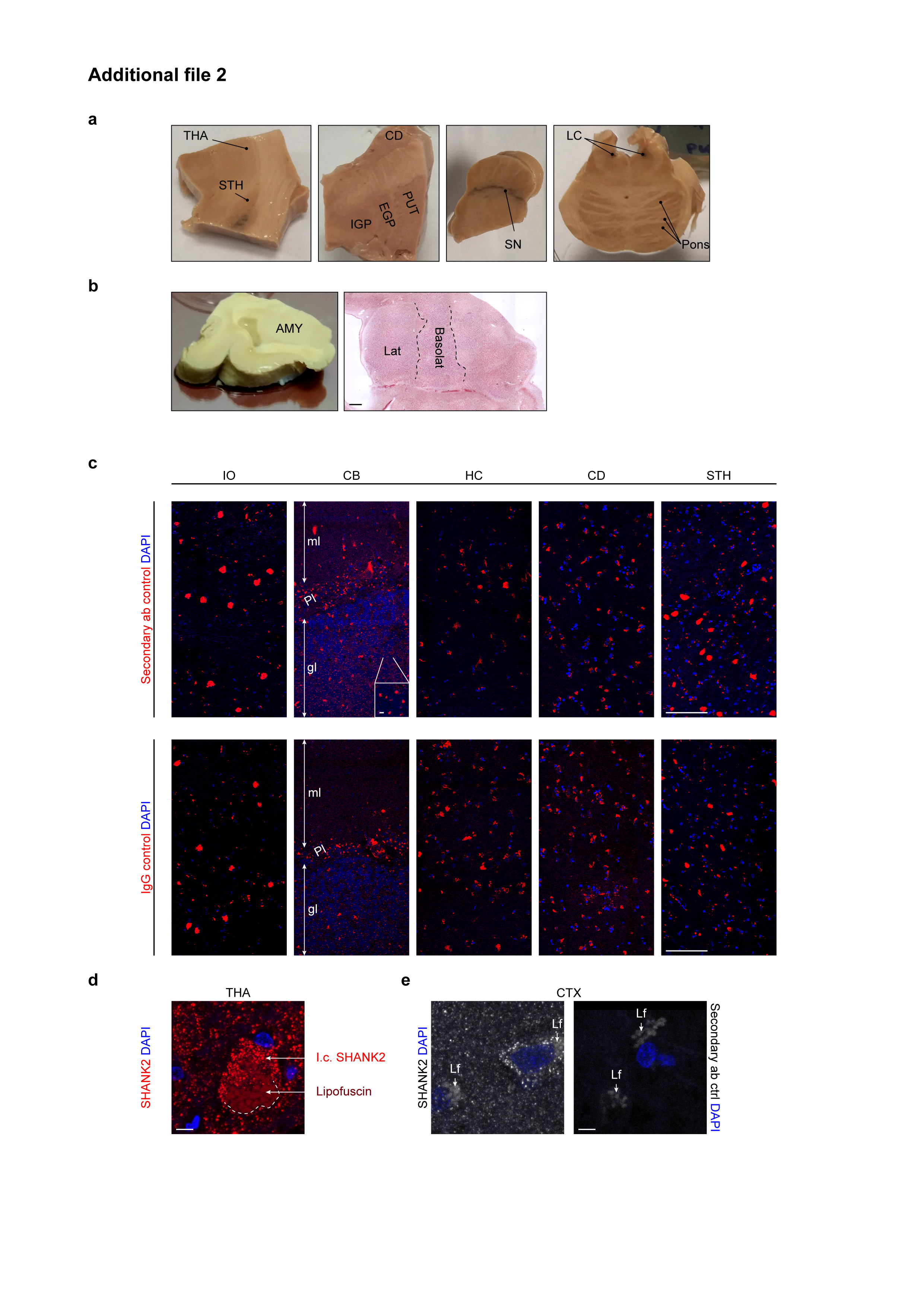

Supplement: Supplementary file 2 — Additional file 2. Negative controls for IF staining of human tissue. a Exemplary photos of dissected human brain regions. b Identification of subregions in the human AMY (left) by pigment Nissl staining (right). Dashed lines indicate the borders of the lateral (lat) and basolateral (basolat) nuclei. Scale bar, 1 mm. c Exemplary images of secondary antibody (ab) and IgG controls of five regions; images for each control were acquired and processed with identical settings. Scale bars, 100 µm, inset CB, 1 µm. ml = molecular layer, Pl = Purkinje cell layer, gl = granular layer. d Intracellular (I.c.) SHANK2 and lipofuscin can be distinguished by their shape, intensity, and distribution (exemplary image of the THA). Scale bar, 5 µm. e SHANK2 staining and secondary ab control (ctrl), acquired in high-resolution with identical settings, image processing identical. Scale bar, 5 µm. Lf = lipofuscin [file 12915_2023_1712_MOESM2_ESM.tif]

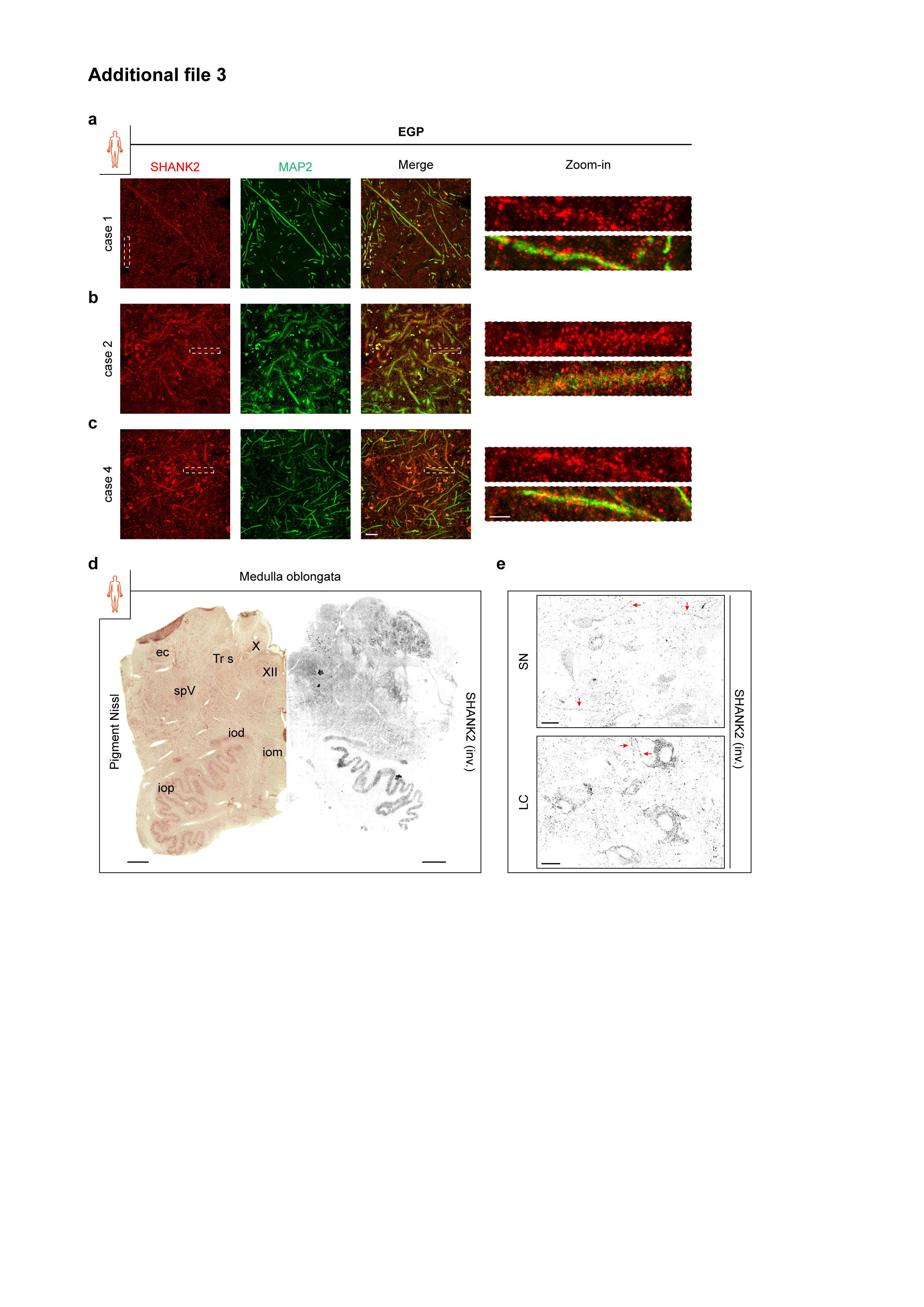

Supplement: Supplementary file 3 — Additional file 3. SHANK2 signal along neurites and somatic SHANK2 in the pallidum, SN, and LC. a-c SHANK2 (left) and MAP2 (middle) co-staining is shown for the single channels and as merge image (right) for cases 1 (a), 2 (b), and 4 (c). A zoom-in from the SHANK2 and merge image (white dashed region) is provided on the outer right side. Images represent maximum intensity projections with z = 5.94 µm. Scale bars, 20 µm (left), 5 µm (zoom-in right). d SHANK2 distribution in a medulla oblongata section from a second case; slightly different cutting level than in Fig. 3c. Nissl staining is shown on the left side, inverted (inv.) SHANK2 IF on the right side. Scale bars, 1 mm. e Inverted (inv.) SHANK2 IF in the SN and LC shows somatic SHANK2 and SHANK2 aligned along neurites (red arrows). Scale bars, 30 µm (SN), 20 µm (LC) [file 12915_2023_1712_MOESM3_ESM.tif]

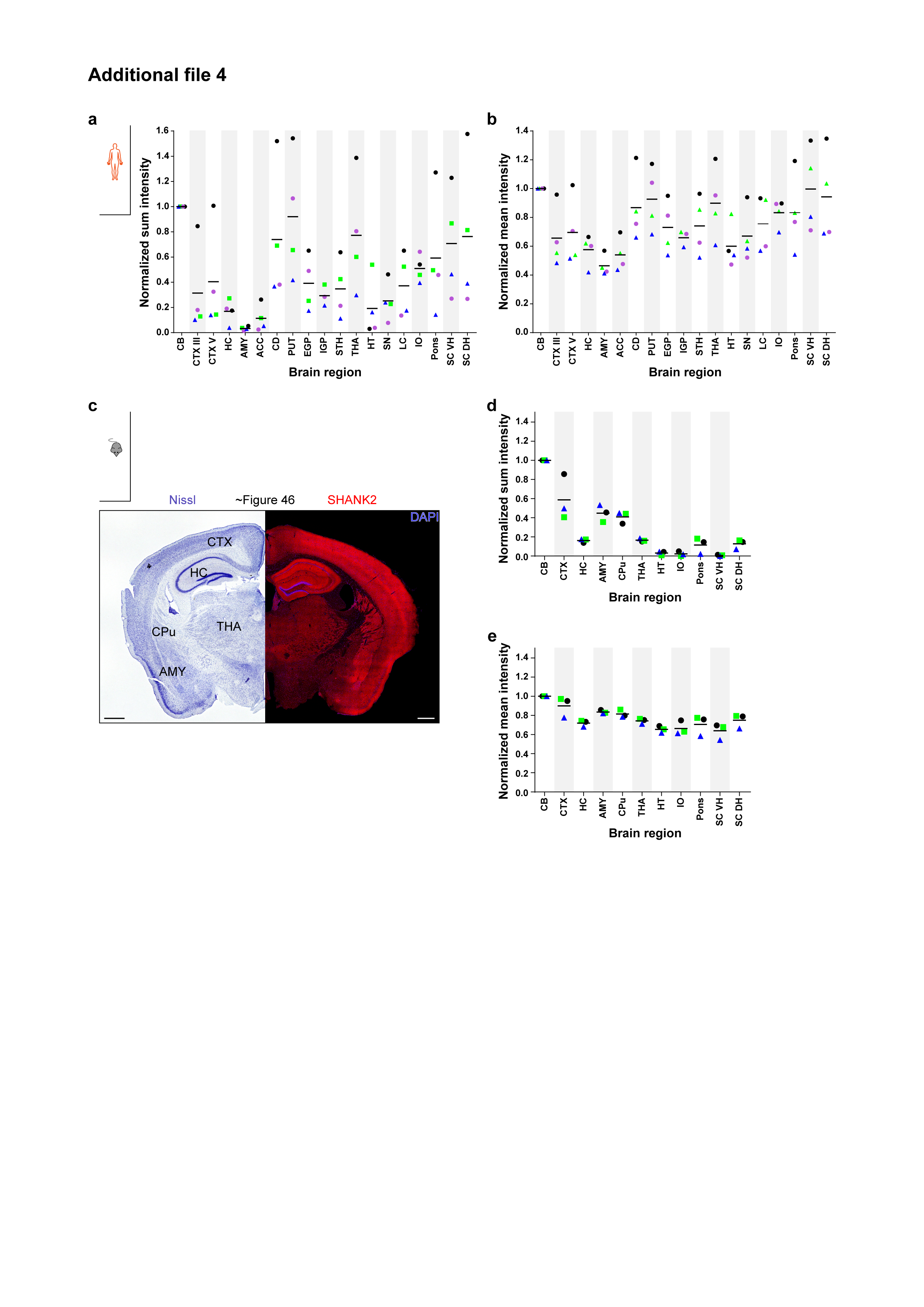

Supplement: Supplementary file 4 — Additional file 4. Sum and mean intensity of SHANK2-positive puncta in the neuropil of human brain regions. a, b Graphs are based on the analysis in Fig. 1b; the normalized sum intensity (a) and mean intensity (b) of SHANK2-positive puncta in the neuropil is plotted (n = 4). c Nissl staining and overview scan of SHANK2 IF in a coronal mouse section. The figure number refers to the used brain atlas (see “Methods”). Scale bars, 500 µm. d, e Graphs are based on the analysis in Fig. 1c; (n = 3), identical to humans in a, b. In a, b and d, e, values were normalized to the CB; means of the analyzed n are indicated by horizontal lines [file 12915_2023_1712_MOESM4_ESM.tif]

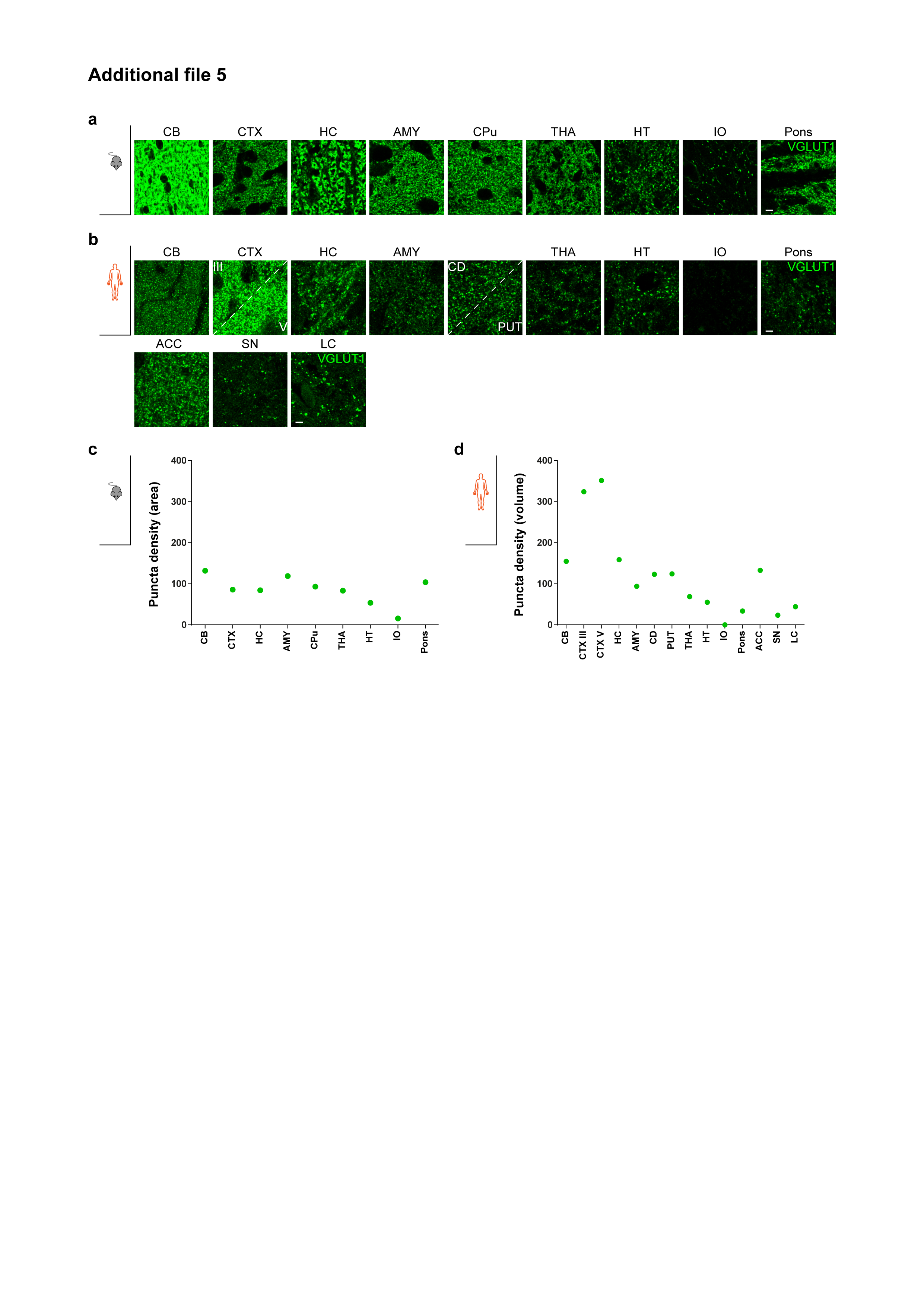

Supplement: Supplementary file 5 — Additional file 5. Synaptic density in human and mouse brain regions as determined by VGLUT1. a, b VGLUT1 IF in most of the mouse (a) and human (b) brain regions included in the SHANK2 analysis (n = 1 each). EGP and IGP were excluded since VGLUT1 protein is not localized in these regions of the human brain [55]. Scale bars, 5 µm. c, d VGLUT1 puncta density for mouse (2D) (c) and human (3D) (d). Owing to an obvious decrease in VGLUT1 intensity from the superficial to lower z-planes in mouse, analysis following deconvolution was performed only in the superficial layer (2D) [file 12915_2023_1712_MOESM5_ESM.tif]

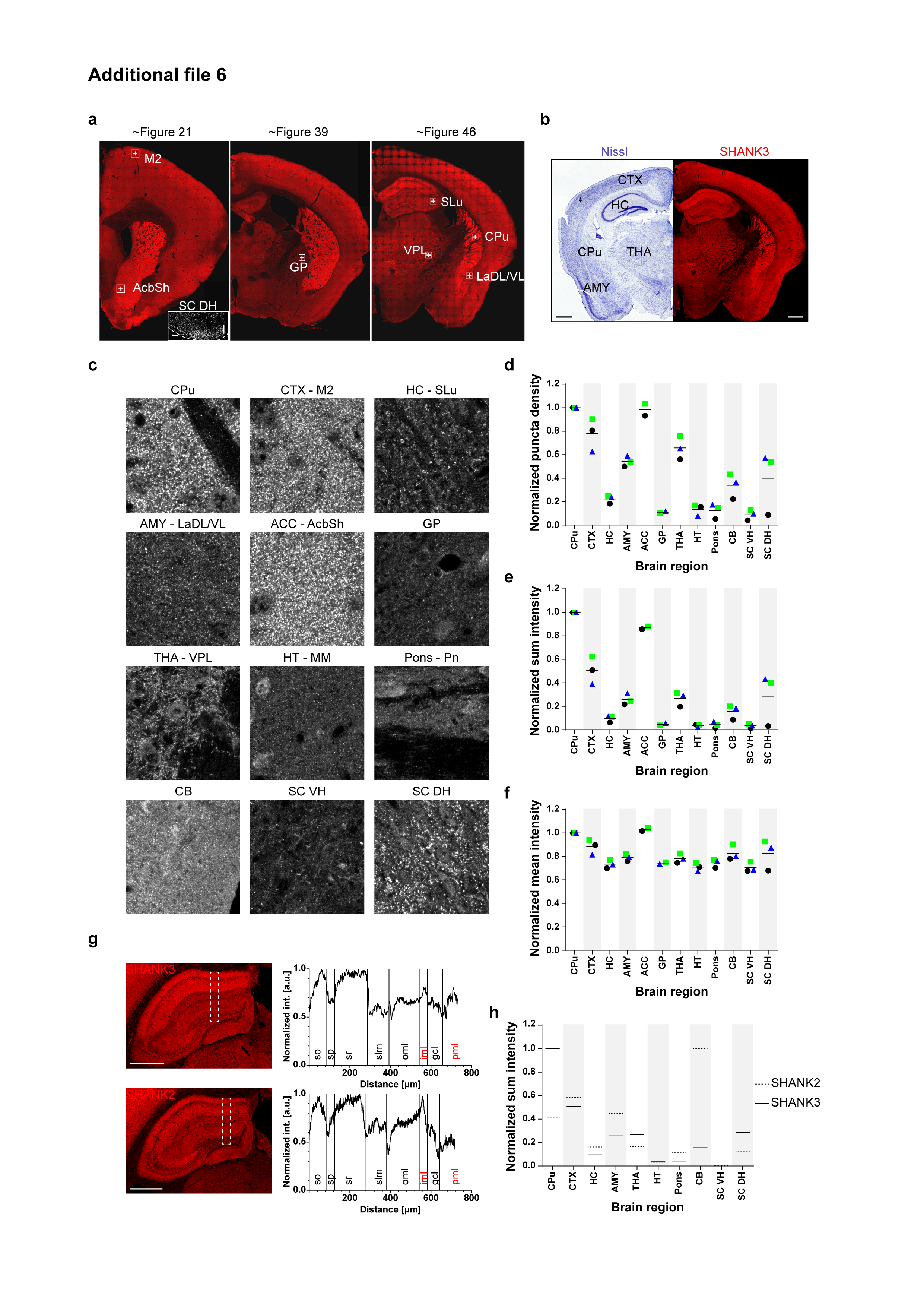

Supplement: Supplementary file 6 — Additional file 6. SHANK3 expression in core mouse brain regions. a Acquisition strategy: Low-resolution overview scans of each coronal mouse section were performed prior to selecting the positions for the high-resolution images (white rectangles with cross). The figure number refers to the used brain atlas (see “Methods”). The small inset in the left image shows the enrichment of SHANK3 in the SC DH lamina I; the dashed line indicates the section border. Scale bar, 50 µm. b Nissl staining and overview scan of SHANK3 IF in a coronal mouse section. Scale bars, 500 µm. c Representative images of mouse SHANK3 protein expression in all analyzed subregions. Brightness/contrast adjustments were performed identically for all regions. Scale bar, 5 µm. Analysis of SHANK3-positive puncta density (d), their sum intensity (e), and their mean intensity (f) is displayed as described for SHANK2 in Fig. 1c (n = 3). For SHANK3 analysis, ACC and GP from two mice were additionally included. In d-f, values were normalized to the CPu; means of the analyzed n are indicated by horizontal lines. g SHANK3 and SHANK2 intensity in the mouse HC. Intensity profiles were generated on raw data (white boxes, 90° rotated). Intensities (int.) were normalized to the maximum values. Scale bars, 500 µm. a.u. = arbitrary unit. h Comparison of SHANK2 and SHANK3 intensity, means were combined from Additional file 4d (SHANK2, dashed line) and Additional file 6e (SHANK3, continuous line) [file 12915_2023_1712_MOESM6_ESM.tif]

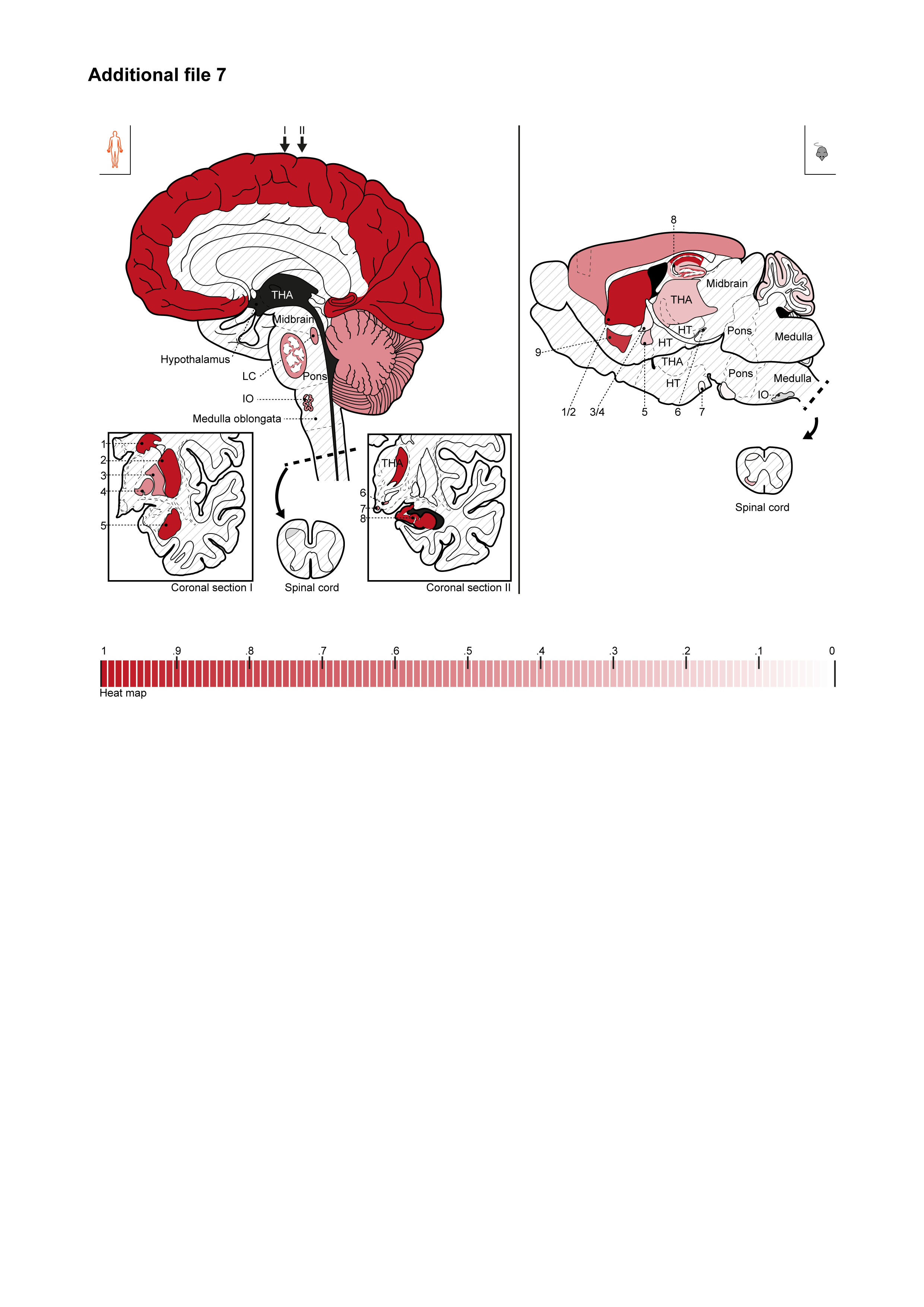

Supplement: Supplementary file 7 — Additional file 7. Heat map of SHANK3 expression in human and analyzed mouse brain regions (neuropil). The sum intensities (mean) of Additional file 6e are represented in the mouse sketches, i.e., color intensities represent SHANK3 expression in the neuropil of each subregion. The applied heat map is shown at the bottom (intervals of 0.01, CPu was dyed with the color intensity corresponding to 1). Information for the human brain was retrieved from [31]. Regions described to stain “pale”, “light(er)”, or “weak” were dyed with the color for 0.5, regions with higher SHANK3 protein expression with the color intensity corresponding to 1. For the CTX and THA (mouse), the color from the analyzed subregion was extrapolated to the entire region since no strong differences were seen in the overview scans. The analyzed subregions are dashed. For the HC (mouse), intensities for all layers were determined by comparing the analyzed region(s) to the non-analyzed layers in overview scans. Overall, crossed out regions were not analyzed; gray regions were only analyzed in the other species. The sagittal sections are based on "Sobotta, Atlas der Anatomie; Kopf, Hals und Neuroanatomie" (24th edition, p. 271) and the used mouse brain atlas (see “Methods”). The coronal sections are based on "The Atlas of the Human Brain". 1 = CD, 2 = PUT, 3 = EGP, 4 = IGP, 5 = AMY (mouse: to present all regions, the extended AMY was colored as substitute), 6 = SN, 7 = HT, 8 = HC, 9 = ACC (see Table S1) [file 12915_2023_1712_MOESM7_ESM.tif]

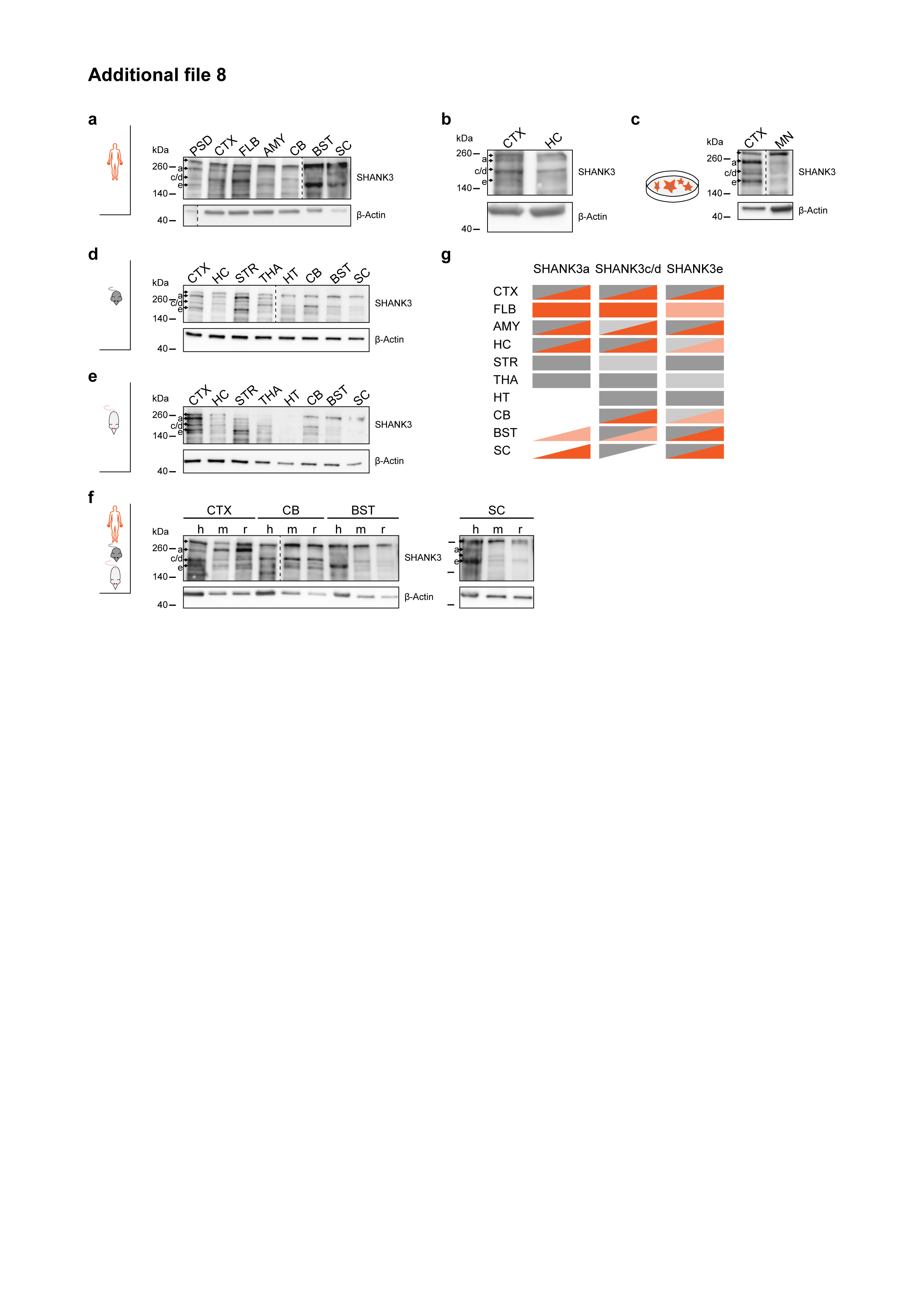

Supplement: Supplementary file 8 — Additional file 8. Conserved SHANK3 isoform expression in human and rodent brain regions. SHANK3 isoform expression in human (a), mouse (d), and rat (e) brain lysates (for d, e: 8% gels). The PSD sample in a was derived from a mouse brain. b SHANK3 isoforms in human CTX and HC lysates (here: 25 µg each, separated in 8% gels), which were used for initial experiments. c SHANK3 isoforms in motor neuron (MN) lysates in comparison to a mouse CTX sample. f The brain regions available from all species were blotted next to each other. Dashed lines indicate different exposure times for the left and right half of the membrane. g Summary of isoform expression in human (orange) and mouse (gray), shading represents expression strength. kDa = kilodalton, unlabeled arrow = 300 kDa band, h = human, m = mouse, r = rat. The uncropped blots are shown in Additional files 11 and 12 [file 12915_2023_1712_MOESM8_ESM.tif]

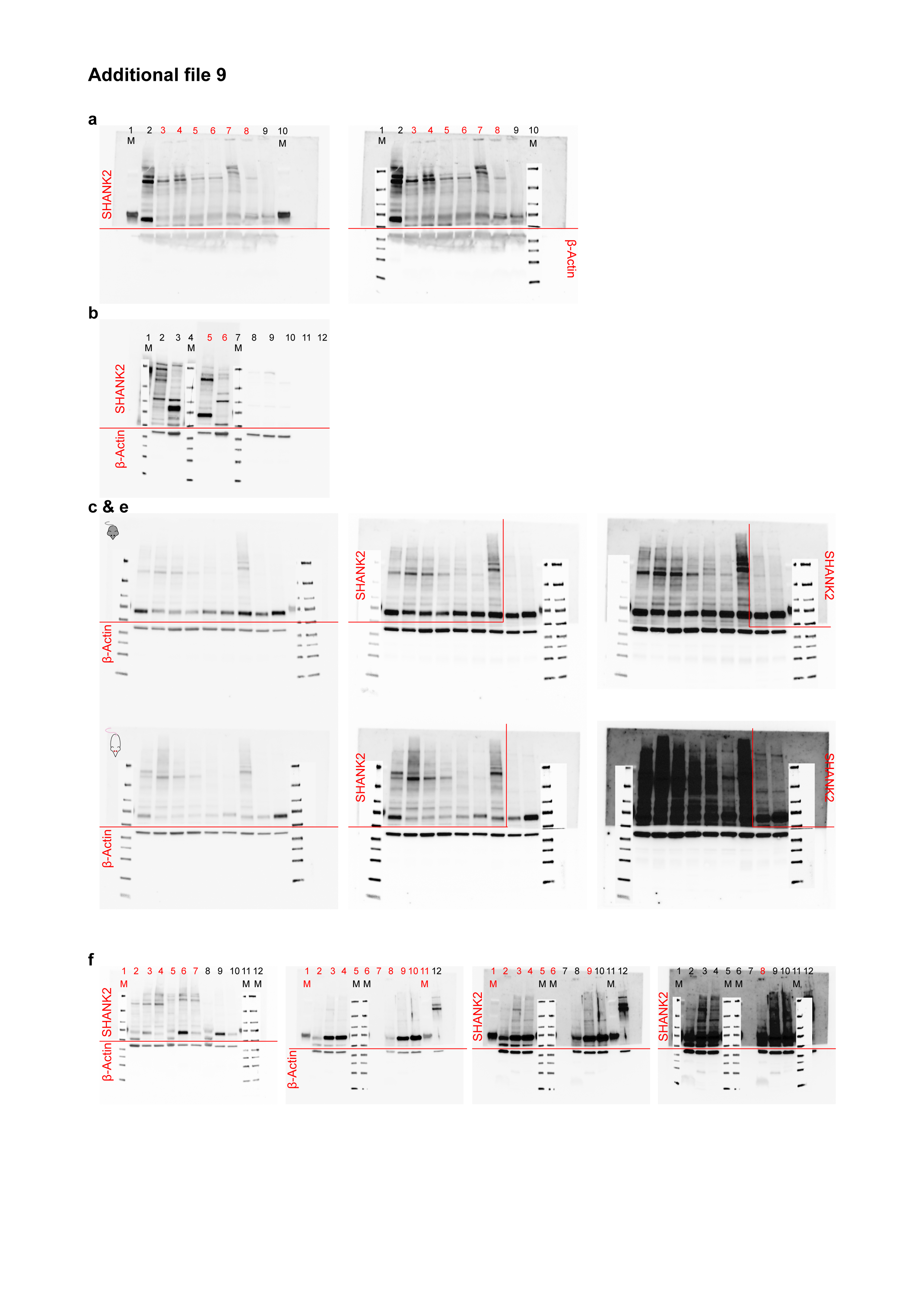

Supplement: Supplementary file 9 — Additional file 9. Raw data for western blots. Uncropped membranes are included as raw data. Membrane cutting before primary antibody incubation is indicated by red lines and those parts of the membrane that are shown in a figure are labeled with the respective antibody. If not the whole part is shown, the numbers of the extracted lanes are highlighted in red. Panel labeling in Additional files 9, 10, 11, and 12 refers to the original figures; the assignment is as follows: Figure 5 → Additional file 9. M = marker [file 12915_2023_1712_MOESM9_ESM.tif]

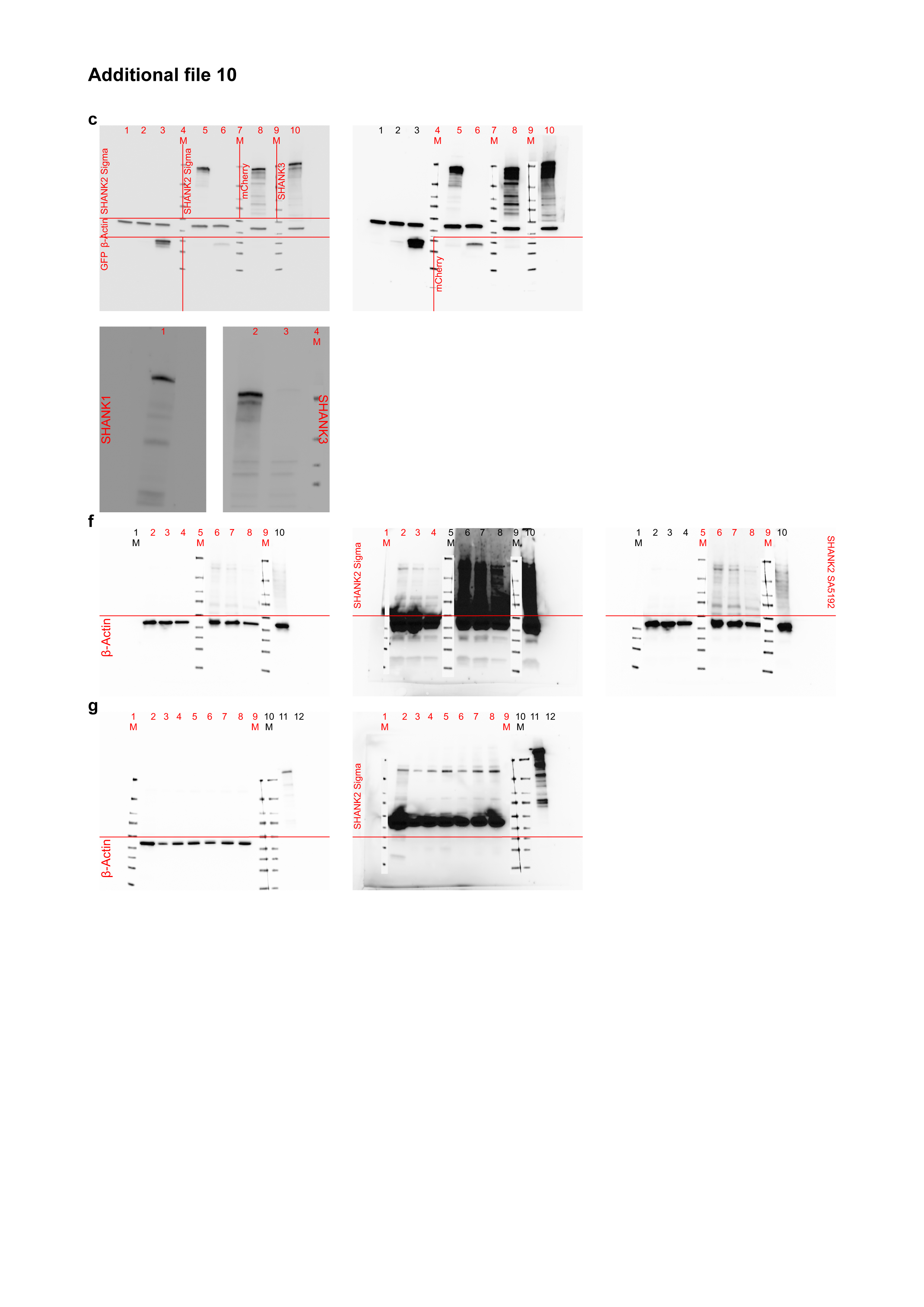

Supplement: Supplementary file 10 — Additional file 10. Raw data for western blots. Uncropped membranes are included as raw data. Membrane cutting before primary antibody incubation is indicated by red lines and those parts of the membrane that are shown in a figure are labeled with the respective antibody. If not the whole part is shown, the numbers of the extracted lanes are highlighted in red. Panel labeling in Additional files 9, 10, 11, and 12 refers to the original figures; the assignment is as follows: Additional file 1 → Additional file 10. M = marker [file 12915_2023_1712_MOESM10_ESM.tif]

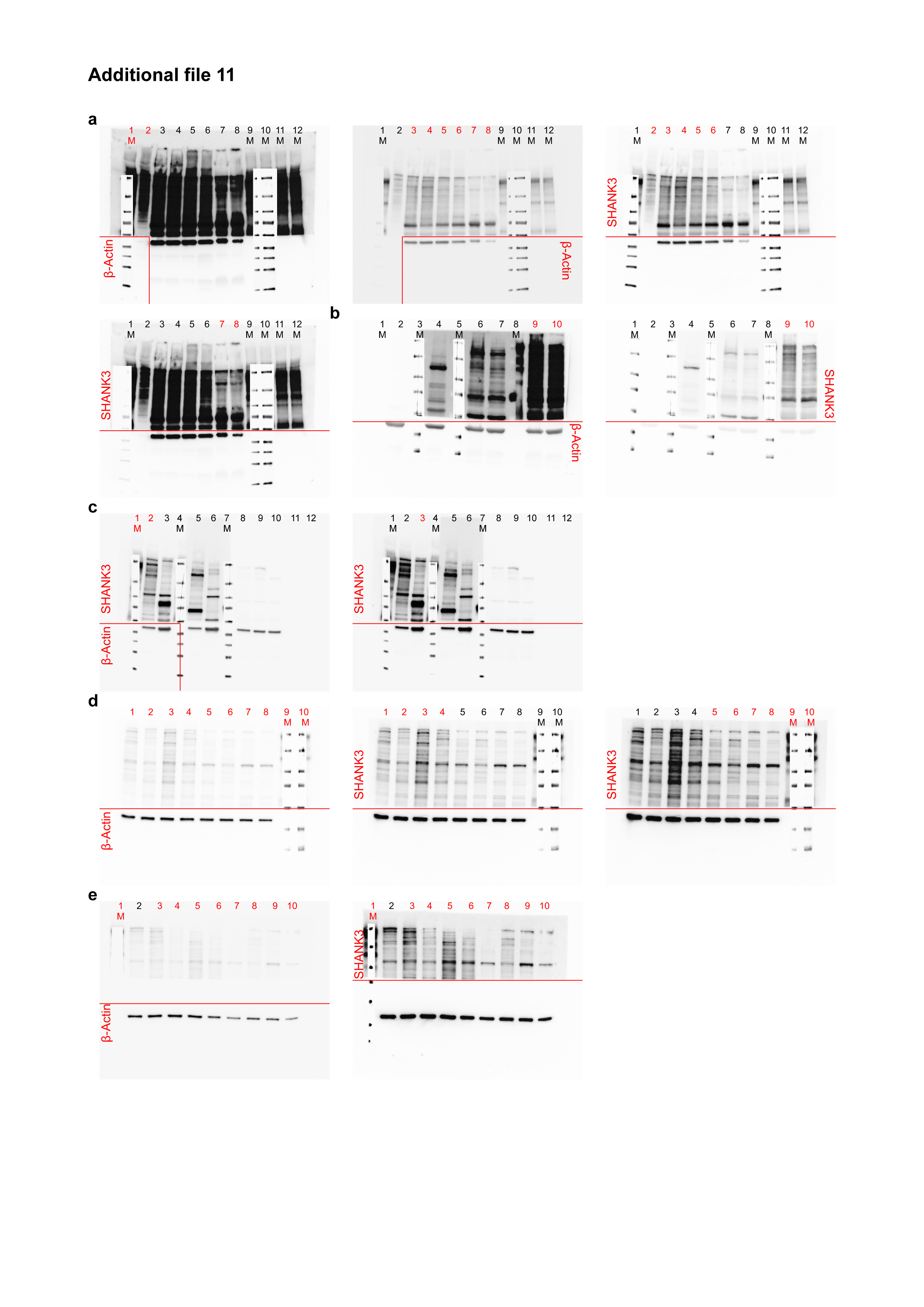

Supplement: Supplementary file 11 — Additional file 11. Raw data for western blots. Uncropped membranes are included as raw data. Membrane cutting before primary antibody incubation is indicated by red lines and those parts of the membrane that are shown in a figure are labeled with the respective antibody. If not the whole part is shown, the numbers of the extracted lanes are highlighted in red. Panel labeling in Additional files 9, 10, 11, and 12 refers to the original figures; the assignment is as follows: Additional file 8 → Additional files 11 and 12. M = marker [file 12915_2023_1712_MOESM11_ESM.tif]

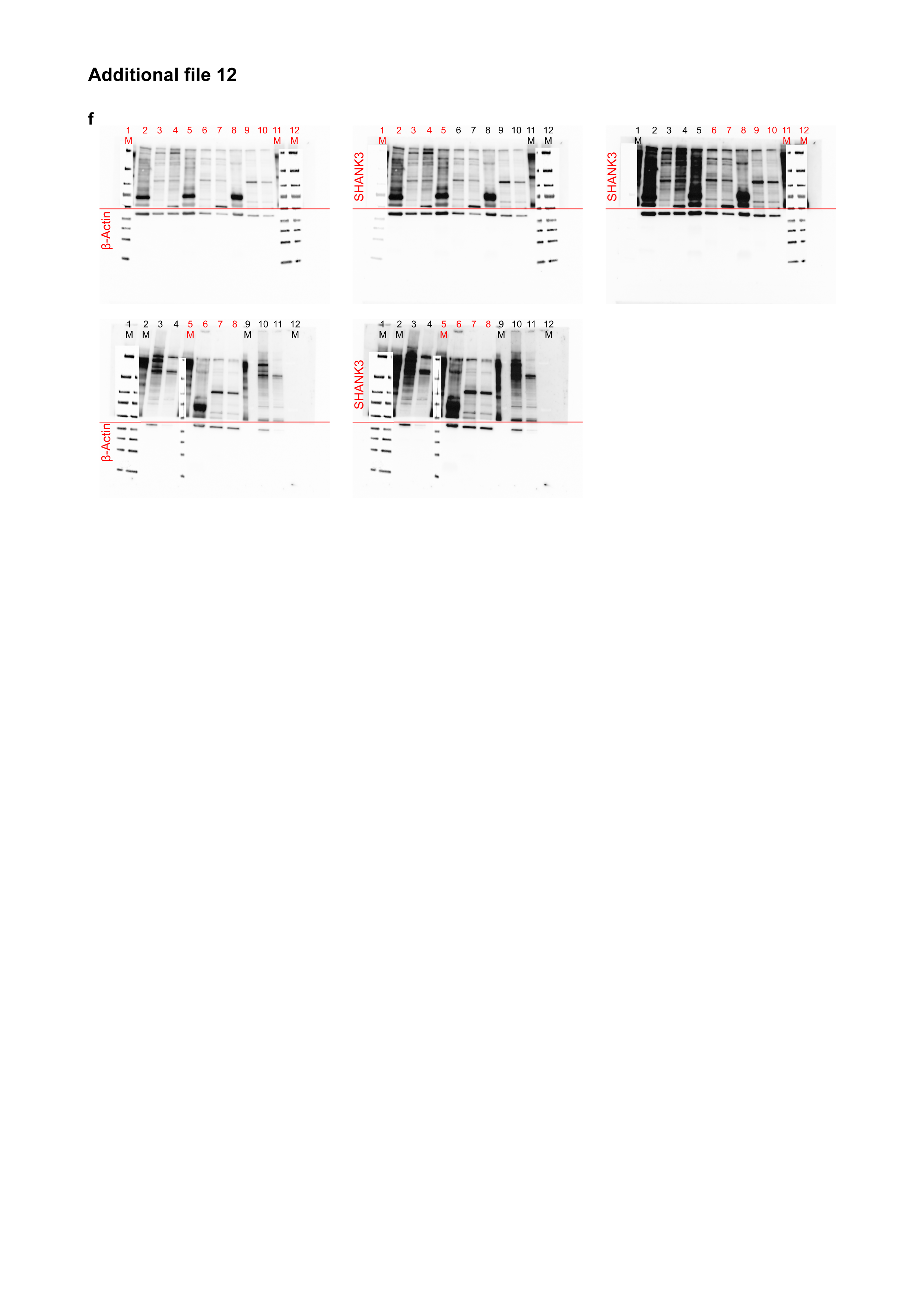

Supplement: Supplementary file 12 — Additional file 12. Raw data for western blots. Uncropped membranes are included as raw data. Membrane cutting before primary antibody incubation is indicated by red lines and those parts of the membrane that are shown in a figure are labeled with the respective antibody. If not the whole part is shown, the numbers of the extracted lanes are highlighted in red. Panel labeling in Additional files 9, 10, 11, and 12 refers to the original figures; the assignment is as follows: Additional file 8 → Additional files 11 and 12. M = marker [file 12915_2023_1712_MOESM12_ESM.tif]
