# Supplementary material for: Expression profiles of the autism-related SHANK proteins in the human brain
Source: BMC Biol. 2023 Nov 13;21:254. doi: 10.1186/s12915-023-01712-0 (PMC10641957; doi:10.1186/s12915-023-01712-0)
Supplement: Supplementary file 13 — Additional file 13: Table S1. Analyzed regions (SHANK2) in human and mouse brains. Table S2. Primary antibodies used for IF staining and western blots. Table S3. Secondary antibodies used for IF staining (not STED) and western blots [file 12915_2023_1712_MOESM13_ESM.docx]

**Table S1** Analyzed regions (SHANK2) in human and mouse brains

| Brain region | Abbrev. | Analyzed subregion / layer | Reference Human | Reference  Mouse |
| --- | --- | --- | --- | --- |
| Cerebral cortex | CTX III  CTX V | Superior frontal gyrus (SFG), layers III & V (human),  Lateral / medial parietal association cortex (mouse) | Fig.: S-01, SFG | Figure 46, LPtA / MPtA |
| Hippocampus | HC | CA3, *stratum pyramidale* (human)  CA3, *stratum lucidum* (mouse) | Plate# 50, CA3 | Figure 46, SLu |
| Amygdala | AMY | Lateral amygdaloid nucleus, dorsal anterior p. (human), lateral amygdaloid nucleus, dorso-/ventrolateral part (mouse) | Plate# 38, LaDA | Figure 46, LaDL / LaVL |
| Accumbens nucleus | ACC | Medial p. | Plate# 25, AcM | N.a. |
| Caudate nucleus | CD | Medial caudate nucleus | Plate# 38, CdM | Figure 46, CPu |
| Putamen | PUT | - | Plate# 38, Pu | See above |
| External globus pallidus | EGP | - | Plate# 38, EGP | N.a. |
| Internal globus pallidus | IGP | - | Plate# 38, IGP | N.a. |
| Subthalamic nucleus | STH | - | Plate# 44, STh | N.a. |
| Thalamus | THA | Lateral ventroanterior n. (human), ventral posterolateral thalamic nucleus (mouse) | Plate# 44, VAL | Figure 46, VPL |
| Hypothalamus | HT | Medial mammillary nucleus, medial p. | Plate# 44, MM | Figure 56, MM |
| Substantia nigra | SN | *Pars compacta* | Plate# 44, SNC | N.a. |
| Locus coeruleus | LC | - | LC | N.a. |
| Inferior olive | IO | Principal subnucleus | IO | ~Figure 89 |
| Pons | Pons | Pontine nuclei | Pons | Figure 67, Pn |
| Cerebellum | CB | Cerebrocerebellum,  Molecular layer | Cb | Figure 77 |
| Spinal cord, lumbar | SC VH | Ventral horn, lamina IX |  |  |
|  | SC DH | Dorsal horn, lamina I |  |  |

Abbrev. = abbreviation, LPtA/MPtA = lateral/medial parietal association cortex, CA = *cornu Ammonis*, p. = part, n.a. = not analyzed, CPu = caudate putamen (striatum). Entire mouse brains were sectioned coronally and sections representing the indicated figures (plates for human) were chosen for analysis (for human and mouse brain atlas, see Methods).

**Table S2** Primary antibodies used for IF staining and western blots

| Primary antibody | Host | Dilution | | RRID |
| --- | --- | --- | --- | --- |
|  |  | Staining | Western blot |  |
| β-Actin | ms | - | 1:10,000/  1:100,000 | Sigma-Aldrich Cat# A5316, RRID:AB_476743 |
| Calbindin D28k | ms | 1:200 | - | Synaptic Systems Cat# 214 011, RRID:AB_2068201 |
| GFP | ms | - | 1:1000 | Takara Bio Cat# 632380, RRID:AB_10013427 |
| GLUA2 | ms | 1:200 | - | Synaptic Systems Cat# 182 111, RRID:AB_10645888 |
| IgG | rb | 1:10,000 | - | Abcam Cat# ab37415, RRID:AB_2631996 |
| MAP2 | ms | 1:200 | - | Millipore Cat# MAB3418, RRID:AB_94856 |
| mCherry | rb | - | 1:2000 | Rockland Cat# 600-401-P16, RRID:AB_2614470 |
| SHANK1 | rb | - | 1:500 | Novus Cat# NB300-166, RRID:AB_10002626 |
| SHANK2 | rb | 1:100 | 1:500  1:300  (**Additional file** 1g) | Sigma-Aldrich Cat# HPA008174, RRID:AB_1856822 |
| SHANK2 | rb | 1:200 | 1:500 | Homemade antiserum  “ppI-SAM pab SA5192” [10] |
| SHANK3 | rb | 1:200 | 1:500 | Homemade antiserum  “PRC pab” [18] |
| SYP | gp | 1:500 | - | Synaptic Systems Cat# 101 004, RRID:AB_1210382 |
| TH | ms | 1:200 | - | Millipore Cat# MAB318, RRID:AB_2201528 |
| VGLUT1 | gp | 1:500 | - | Synaptic Systems Cat# 135 304, RRID:AB_887878 |

Ms = mouse, rb = rabbit, gp = guinea pig.

**Table S3** Secondary antibodies used for IF staining (not STED) and western blots

| Secondary antibody | Host | Dilution | | RRID |
| --- | --- | --- | --- | --- |
|  |  | Staining | Western blot |  |
| Alexa Fluor 647  anti-rabbit | goat | 1:200 | - | Thermo Fisher Scientific  Cat# A-21245, RRID:AB_2535813 |
| Alexa Fluor 647  anti-rabbit | donkey | 1:200 | - | Jackson ImmunoResearch Labs Cat# 711-605-152, RRID:AB_2492288 |
| Alexa Fluor 647  anti-guinea pig | donkey | 1:200 | - | Jackson ImmunoResearch Labs Cat# 706-605-148, RRID:AB_2340476 |
| Alexa Fluor 594  anti-mouse | donkey | 1:200 | - | Jackson ImmunoResearch Labs Cat# 715-585-151, RRID:AB_2340855 |
| Alexa Fluor 546  anti-mouse | goat | 1:200 | - | Thermo Fisher Scientific  Cat# A-11030, RRID:AB_2534089 |
| Alexa Fluor 488  anti-mouse | goat | 1:200 | - | Thermo Fisher Scientific  Cat# A-11029, RRID:AB_2534088 |
| Alexa Fluor 488  anti-guinea pig | goat | 1:200 | - | Thermo Fisher Scientific  Cat# A-11073, RRID:AB_2534117 |
| Alexa Fluor 488  anti-guinea pig | donkey | 1:200 | - | Jackson ImmunoResearch Labs Cat# 706-545-148, RRID:AB_2340472 |
| Polyclonal anti-rabbit Ig/HRP antibody | goat | - | 1:1000 | Agilent Cat# P0448, RRID:AB_2617138 |
| Polyclonal anti-mouse Ig/HRP antibody | rabbit | - | 1:3000 | Agilent Cat# P0260, RRID:AB_2636929 |
